# Supplementary material for: A metal-trap tests and refines blueprints to engineer cellular protein metalation with different elements
Source: Nat Commun. 2025 Jan 18;16:810. doi: 10.1038/s41467-025-56199-w (PMC11742986; doi:10.1038/s41467-025-56199-w)
Supplement: Supplementary file 1 — Supplementary Information [file 41467_2025_56199_MOESM1_ESM.pdf]

**Supplementary information for**

# A metal-trap tests and refines blueprints to engineer cellular protein metalation with different elements

Sophie E. Clough[1,2,†], Tessa R. Young[1,2,†], Emma Tarrant[1,2], Andrew J.P. Scott[1,2], Peter T. Chivers[1,2], Arthur Glasfeld[1,2], and Nigel J. Robinson[1,2*]

[1]Department of Biosciences, University of Durham, UK

[2]Department of Chemistry, University of Durham, UK

[†]These authors contributed equally: Sophie E. Clough, Tessa R. Young

*e-mail: nigel.robinson@durham.ac.uk

**Table of contents**

**Supplementary Table 1.** Compositions of solutions used for *in vitro* folding of MncA with competing metal ions shown in Figure 1b.

**Supplementary Table 2.** Trials conducted to test trapping preference for $Ni^{II}$ in unbuffered solutions versus $Mn^{II}$ or $Co^{II}$.

**Supplementary Table 3.** Crystallographic data collection and refinement statistics for $(Ni^{II})_2$MncA.

**Supplementary Table 4.** Metal contents and percentage occupancies of *in vivo* metalated MncA containing fractions isolated from *E. coli*.

**Supplementary Table 5.** Metal contents and percentage occupancies of *in vivo* metalated MncA containing fractions isolated from *E. coli* in 4 mM manganese.

**Supplementary Table 6.** Metal contents and percentage occupancies of *in vivo* metalated MncA containing fractions isolated from *E. coli* in 800 μM zinc.

**Supplementary Table 7.** Metal contents and percentage occupancies of *in vivo* metalated MncA containing fractions isolated from *E. coli* in 600 μM copper.

**Supplementary Table 8.** Metal contents and percentage occupancies of *in vivo* metalated MncA containing fractions isolated from *E. coli* in 600 μM nickel.

**Supplementary Table 9.** Metal contents and percentage occupancies of *in vivo* metalated MncA containing fractions isolated from *E. coli* in 300 μM cobalt.

**Supplementary Fig. 1.** A model for the speciation of metalation.

**Supplementary Fig. 2.** Refolding MncA to establish relative preference for $Ni^{II}$ and competition with BCA-buffered $Cu^{I}$.

**Supplementary Fig. 3.** Characterisation of $Ni^{II}$-RcnR.

**Supplementary Fig. 4.** Calibrated metal sensor responses as a function of intracellular metal availability.

**Supplementary Fig. 5.** Relative metal preferences decode metal speciation of MncA and refined mid-range availabilities decode cognate metals of five proteins.

**Supplementary Fig. 6.** Omit electron density maps of $(Ni^{II})_2$MncA showing metal coordination environments.

**Supplementary Fig. 7.** $Ni^{II}$ is kinetically trapped by MncA.

**Supplementary Fig. 8.** SDS-PAGE of MncA purification in soluble form following expression in *E. coli*.

**Supplementary Fig. 9.** Effects of elevated metals on growth of *E. coli*.

**Supplementary Fig. 10.** SDS-PAGE of MncA purified in soluble form following expression in *E. coli* in 4 mM manganese.

**Supplementary Fig. 11.** SDS-PAGE and exemplar chromatogram of MncA purified in soluble form following expression in *E. coli* in 800 μM zinc.

**Supplementary Fig. 12.** SDS-PAGE and exemplar chromatogram of MncA purified from *E. coli* in 600 μM copper.

**Supplementary Fig. 13.** SDS-PAGE and exemplar chromatogram of MncA further purified from *E. coli* in 600 μM copper with Blue Sepharose.

**Supplementary Fig. 14.** SDS-PAGE and exemplar chromatogram of MncA purified in soluble form following expression in *E. coli* in 600 μM nickel.

**Supplementary Fig. 15.** SDS-PAGE and exemplar chromatogram of MncA purified in soluble form following expression in *E. coli* in 300 μM cobalt.

**Supplementary Fig. 16.** MncA-refined metal availabilities for *E. coli* in metal-supplemented medium.

**Supplementary Fig. 17.** ICP-MS shows MntR-independent decline in total manganese atoms $cell^{-1}$ in high nickel, and Fur-independent decline in total iron in high manganese.

**Supplementary Fig. 18** Shallow $Mn^{II}$ pool depleted after 18 h MncA expression in un-supplemented media.

**Supplementary Fig. 19** Larger gradients ($\Delta\Delta G$) for $Fe^{II}$ and $Mn^{II}$ predicted to inhibit metalation of MncA with $Zn^{II}$ at elevated $Zn^{II}$ availability (99% activation of ZntR).

**Supplementary Fig. 20.** Predicted mis-metalation of non-native proteins expressed in *E. coli*.

**Supplementary Note 1.** Derivation of calculations to formulate metal buffers with defined availabilities of two competing metals (Supplementary Data 1).

**Supplementary Note 2.** Derivation of calculations to refine intracellular metal availabilities using MncA as a probe (Supplementary Data 5).

## Supplementary References

**Supplementary Table 1.** Compositions of solutions used for *in vitro* folding of MncA with competing metal ions shown in Figure 1c.

| [a]Metal 1 | Metal 2 | Buffer | [b]$[M1]_{total}$ | $[M2]_{total}$ | $[buffer]_{total}$ | $[M1]_{free}$ | $[M2]_{free}$ | $\frac{[M1]_{free}}{[M2]_{free}}$ |
|---|---|---|---|---|---|---|---|---|
| $Mn^{II}$ | $Fe^{II}$ | NTA | $1.78 \times 10^{-4}$ | $2.10 \times 10^{-3}$ | $3.00 \times 10^{-3}$ | $2.22 \times 10^{-6}$ | $6.20 \times 10^{-7}$ | 3.58 |
| $Mn^{II}$ | $Fe^{II}$ | NTA | $1.70 \times 10^{-5}$ | $3.10 \times 10^{-5}$ | $3.00 \times 10^{-4}$ | $6.03 \times 10^{-7}$ | $2.67 \times 10^{-8}$ | 22.6 |
| $Mn^{II}$ | $Fe^{II}$ | NTA | $6.70 \times 10^{-4}$ | $1.10 \times 10^{-3}$ | $2.00 \times 10^{-3}$ | $2.20 \times 10^{-5}$ | $8.50 \times 10^{-7}$ | 25.9 |
| $Mn^{II}$ | $Co^{II}$ | NTA | $1.00 \times 10^{-5}$ | $5.00 \times 10^{-4}$ | $7.50 \times 10^{-4}$ | $3.73 \times 10^{-7}$ | $1.48 \times 10^{-8}$ | 25 |
| $Mn^{II}$ | $Co^{II}$ | NTA | $5.60 \times 10^{-5}$ | $6.98 \times 10^{-4}$ | $1.00 \times 10^{-3}$ | $2.00 \times 10^{-6}$ | $2.00 \times 10^{-8}$ | 100 |
| $Mn^{II}$ | $Co^{II}$ | NTA | $1.16 \times 10^{-4}$ | $3.67 \times 10^{-4}$ | $1.00 \times 10^{-3}$ | $2.00 \times 10^{-6}$ | $5.00 \times 10^{-9}$ | 400 |
| $Mn^{II}$ | $Ni^{II}$ | His | $1.00 \times 10^{-5}$ | $3.00 \times 10^{-4}$ | $1.00 \times 10^{-3}$ | $9.80 \times 10^{-6}$ | $8.50 \times 10^{-10}$ | 11529 |
| $Mn^{II}$ | $Ni^{II}$ | His | $1.00 \times 10^{-5}$ | $2.00 \times 10^{-4}$ | $1.00 \times 10^{-3}$ | $9.70 \times 10^{-6}$ | $2.60 \times 10^{-10}$ | [c]37308 |
| $Mn^{II}$ | $Ni^{II}$ | His | $1.00 \times 10^{-5}$ | $4.00 \times 10^{-4}$ | $1.00 \times 10^{-3}$ | $9.90 \times 10^{-6}$ | $4.10 \times 10^{-9}$ | 2415 |
| $Mn^{II}$ | $Ni^{II}$ | His | $1.00 \times 10^{-5}$ | $3.50 \times 10^{-4}$ | $1.00 \times 10^{-3}$ | $9.85 \times 10^{-6}$ | $1.72 \times 10^{-9}$ | 5727 |
| $Mn^{II}$ | $Zn^{II}$ | NTA | $4.10 \times 10^{-4}$ | $5.41 \times 10^{-5}$ | $1.00 \times 10^{-3}$ | $6.80 \times 10^{-6}$ | $3.72 \times 10^{-10}$ | 18280 |
| $Mn^{II}$ | $Zn^{II}$ | NTA | $6.39 \times 10^{-5}$ | $8.48 \times 10^{-5}$ | $1.00 \times 10^{-3}$ | $6.80 \times 10^{-7}$ | $3.70 \times 10^{-10}$ | 1838 |
| $Mn^{II}$ | $Zn^{II}$ | NTA | $3.65 \times 10^{-5}$ | $4.81 \times 10^{-4}$ | $1.00 \times 10^{-3}$ | $6.80 \times 10^{-7}$ | $3.72 \times 10^{-9}$ | 183 |
| $Mn^{II}$ | $Cu^{II}$ | NTA | $1.05 \times 10^{-4}$ | $7.71 \times 10^{-4}$ | $1.00 \times 10^{-3}$ | $6.80 \times 10^{-6}$ | $1.00 \times 10^{-10}$ | 68000 |
| $Mn^{II}$ | $Cu^{II}$ | NTA | $5.88 \times 10^{-4}$ | $4.10 \times 10^{-4}$ | $1.00 \times 10^{-3}$ | $6.80 \times 10^{-5}$ | $1.00 \times 10^{-10}$ | 680000 |
| $Mn^{II}$ | $Cu^{II}$ | NTA | $8.92 \times 10^{-4}$ | $6.50 \times 10^{-5}$ | $1.00 \times 10^{-3}$ | $6.80 \times 10^{-5}$ | $1.00 \times 10^{-11}$ | 6800000 |
| $Zn^{II}$ | $Cu^{I}$ | | $9.70 \times 10^{-6}$ | $6.60 \times 10^{-6}$ | | $9.70 \times 10^{-6}$ | $6.60 \times 10^{-6}$ | 1.47 |
| $Zn^{II}$ | $Cu^{I}$ | | $1.12 \times 10^{-5}$ | $1.37 \times 10^{-5}$ | | $1.12 \times 10^{-5}$ | $1.37 \times 10^{-5}$ | 0.82 |
| $Zn^{II}$ | $Cu^{I}$ | | $1.13 \times 10^{-5}$ | $9.85 \times 10^{-6}$ | | $1.13 \times 10^{-5}$ | $9.85 \times 10^{-6}$ | 1.15 |

[a] Buffer compositions were calculated using Supplementary Data 1 or for histidine, *HySS* software[1].
[b] All concentrations are in units of molarity.
[c] A fourth trial competing $Mn^{II}$ against $Ni^{II}$ is not shown in Figure 1 and the chromatograms are in Supplementary Figure 2. Notably, this result is included in the calculated preference for $Ni^{II}$ ($n$ = 4 experimental replicates). Otherwise $n$ = 3 independent replicates are shown. M1 and M2 represent metal 1 and metal 2 as shown.

**Supplementary Table 2.** Trials conducted to test trapping preference for $Ni^{II}$ in unbuffered solutions versus $Mn^{II}$ or $Co^{II}$.

| [M1] | [M1] (M) | [$Ni^{II}$] (M) | [M1]/[$Ni^{II}$] | %M1 | %$Ni^{II}$ | %M1/%$Ni^{II}$ | Preference $Ni^{II}/Mn^{II}$ |
|---|---|---|---|---|---|---|---|
| $Mn^{II}$ | $1.00x10^{-3}$ | $1.00x10^{-5}$ | 100 | 14.3 | 85.7 | 0.17 | 599 |
| $Co^{II}$ | $1.00x10^{-5}$ | $1.00x10^{-5}$ | 1 | 18 | 82 | 0.22 | 665[a] |
| $Co^{II}$ | $1.00x10^{-4}$ | $1.00x10^{-5}$ | 10 | 45.5 | 54.5 | 0.83 | 1750[a] |

[a] In trials where $Co^{II}$ was used as a competitor against $Ni^{II}$, preference for $Ni^{II}$ versus $Mn^{II}$ was obtained by multiplying the experimentally determined preference for $Ni^{II}$ versus $Co^{II}$ by 147, the preference for $Co^{II}$ over $Mn^{II}$. Unbuffered trials were initially attempted because a suitable NTA-based metal buffer system was not possible. Subsequently a histidine-based metal buffer system was used as in Figure 1c and Table 1, generating a 7-fold greater preference for $Ni^{II}$ potentially because metal-buffering overcomes inaccuracies generated by trace competitors from inclusion bodies. *n* = 3 independent replicates are shown.

**Supplementary Table 3.** Crystallographic data collection and refinement statistics for $(Ni^{II})_2$MncA[a]

| **Data Collection Statistics** | |
|---|---|
| Wavelength (Å) | 0.9795 |
| Resolution range (Å) | 76.98 - 1.60 (1.63 - 1.60) |
| Space group | P 65 2 2 |
| Unit cell *a, b, c* axes (Å) | 235.2, 235.2, 132.0 |
| Total reflections | 44288450 (1526344) |
| Unique reflections | 280693 (15191) |
| Multiplicity | 158 (100) |
| Completeness (%) | 99.9 (98.4) |
| Mean $I/\sigma(I)$ | 16.4 (0.54) |
| Wilson *B*-factor | 22.24 |
| $R_{meas}$ (%) | 26.7 (458) |
| $R_{pim}$ (%) | 2.0 (44.4) |
| $CC_{1/2}$ | 1.00 (0.63) |

| **Refinement Statistics** | |
|---|---|
| Reflections used in refinement | 277977 (15028) |
| Reflections used for $R_{free}$ | 14062 (742) |
| $R_{work}$ (%) | 16.3 (40.7) |
| $R_{free}$ (%) | 17.6 (41.9) |
| Number of non-hydrogen atoms | 9298 |
| protein | 8321 |
| ligands | 30 |
| solvent | 947 |
| Protein residues | 1069 |
| RMS (bonds, Å) | 0.006 |
| RMS (angles, °) | 0.87 |
| Ramachandran favoured (%) | 98.3 |
| Ramachandran allowed (%) | 1.7 |
| Ramachandran outliers (%) | 0 |
| Rotamer outliers (%) | 0.34 |
| Clashscore | 1.04 |
| Average *B*-factor | 24.8 |
| macromolecules | 24.0 |
| ligands | 31.2 |
| solvent | 32.1 |
| Number of TLS groups | 16 |

[a] Statistics for the highest-resolution shell are shown in parentheses.

**Supplementary Table 4:** Metal contents and percentage occupancies of *in vivo* metalated MncA containing fractions isolated from *E. coli* [a].

**a.**

| Replicate and (fraction) | [Mn] (µM) | [Fe] (µM) | [Co] (µM) | [Ni] (µM) | [Zn] (µM) | [Cu] (µM) | [MncA] (µM) |
|---|---|---|---|---|---|---|---|
| 1(2)[c] | 0.17 | 3.73 | 0.04 | 0.04 | 0.60 | 0.24 | 2.22 |
| 2(9)[d] | 2.39 | 24.70 | 0.05 | 0.10 | 0.26 | 0.04 | 13.49 |
| 2(10)[d] | 4.45 | 48.67 | 0.10 | 0.22 | 0.72 | 0.03 | 25.65 |
| 3(14)[e] | 0.40 | 3.73 | 0.01 | 0.02 | 0.00[b] | 0.08 | 2.56 |
| 3(15)[e] | 0.58 | 5.82 | 0.01 | 0.01 | 0.04 | 0.03 | 3.68 |
| 4(12)[f] | 0.55 | 7.52 | 0.01 | 0.00[b] | 0.00[b] | 0.00[b] | n/a |
| 4(13)[f] | 1.06 | 14.31 | 0.03 | 0.00[b] | 0.00[b] | 0.00[b] | n/a |
| 4(14)[f] | 0.36 | 5.00 | 0.01 | 0.00[b] | 0.00[b] | 0.00[b] | n/a |

[a] Total metal determined by ICP-MS from analysis of fractions eluting from 1 mL anion exchange column (Supplementary Fig. 8 for corresponding SDS-PAGE analyses). Total protein in each fraction was estimated from absorbance at 280 nm. *n* = 3 independent and analytically identical biological replicates are shown.
[b] Where ICP-MS returned negative values (<0.86 for zinc representing consistently reduced baseline values for this element) these have been reported as zero.
[c] Supplementary Fig. 8e
[d] Supplementary Fig. 8d
[e] Supplementary Fig. 8a-c
[f] Replicate 4 (*n* = 1) used simplified analytical chromatography excluding a second anion exchange step and replacing preparative SEC with more rapid HPLC (TSK SW3000). Supplementary Fig. 8f-g

**b.**

| Replicate | Mn | Fe | Co | Ni | Zn | Cu | Stoichiometry |
|---|---|---|---|---|---|---|---|
| 1 | 5 | 84 | 1 | 1 | 14 | 5 | 109[h] |
| 2[g,k] | 9 | 93 | 0 | 0 | 1 | 0 | 104[h] |
| 3[g] | 8 | 76 | 0 | 0 | 0 | 1 | 85[h] |
| Mean[h] | 7±2 | 84±7 | 0±0 | 0.5±0.2 | 5±6 | 2±2 | 99±10 (≈ 2:1)[h] |
| 1 | 4 | 77 | 1 | 1 | 13 | 5 | n/a |
| 2[a] | 8 | 90 | 0 | 0 | 1 | 0 | n/a |
| 3[a] | 9 | 89 | 0 | 0 | 0 | 1 | n/a |
| Mean[i] | 7±3 | 85±7 | 0±0 | 1±0.3[l] | 5±7 | 2±3 | n/a |
| 4[j] | 7 | 93 | 0 | 0.0 | 0.0 | 0.0 | n/a |

[g] Percent metalation for replicates 2 and 3 reflect averages from two fractions as shown in Supplementary Table 4a.
[h] Percent occupancy of MncA with each metal was calculated from the ratio of [metal]/[MncA]. Total occupancy of MncA sites was calculated assuming 2 metal sites per MncA molecule.
[i] Percent metalation with each metal was calculated from the ratio of [metal]/[total metal]. Total metal concentration is the sum of all metal concentrations obtained in the trial. Similar percentage occupancies were obtained using both approaches because the ratio of metal to MncA was close to 2:1 (equivalent to 100%). Variation in extinction coefficient with different metals, and purity with simplified extraction procedures, means that percentage occupancy in subsequent experiments were calculated as function of total metal rather than estimated [MncA] (*n* = 3 biological replicates ± SD).
[j] Replicate 4 (*n* = 1) used a simplified analytical chromatography excluding a second anion exchange step and replacing preparative SEC with smaller scale and more rapid HPLC (TSK SW3000).
[k] Fig. 4b
[l] Means ± SD were calculated prior to rounding.

**Supplementary Table 5:** Metal contents and percentage occupancies of *in vivo* metalated MncA containing fractions isolated from *E. coli* in 4 mM manganese [a].

**a.**

| Replicate and (fraction) | [Mn] (μM) | [Fe] (μM) | [Co] (μM) | [Ni] (μM) | [Zn] (μM) | [Cu] (μM) |
|---|---|---|---|---|---|---|
| 1(17)[c] | 7.67 | 7.25 | 0.07 | 0.50 | 0.00 | 0.12 |
| 1(18)[c] | 4.80 | 3.93 | 0.04 | 0.48 | 0.00 | 0.18 |
| 2(13)[d] | 11.44 | 4.21 | 0.00 | 0.53 | 0.00 | 0.56 |
| 2(14)[d] | 10.36 | 3.69 | 0.00 | 0.54 | 0.02 | 0.39 |
| 2(15)[d] | 2.82 | 0.95 | 0.00 | 0.53 | 0.09 | 0.39 |
| 3(19)[e] | 6.47 | 2.59 | 0.00 | 0[b] | 0.00 | 0.33 |
| 3(20)[e] | 13.59 | 5.40 | 0.00 | 0[b] | 0.00 | 0.25 |
| 3(21)[e] | 7.13 | 2.83 | 0.00 | 0[b] | 0.29 | 0.25 |

[a] Total metal determined by ICP-MS from analysis of fractions eluting from SEC (TSK SW3000) (Supplementary Fig. 10 for corresponding SDS-PAGE analyses). *n* = 3 independent biological replicates are shown.
[b] Where ICP-MS returned negative values (<0.03) these have been reported as zero.
[c] Supplementary Fig. 10a
[d] Supplementary Fig. 10b
[e] Supplementary Fig. 10c

**b.**

| Replicate | Mn | Fe | Co | Ni | Zn | Cu |
|---|---|---|---|---|---|---|
| 1[f] | 50 | 44 | 0 | 4 | 0 | 1 |
| 2[f] | 65 | 23 | 0 | 1 | 1 | 5 |
| 3[f,g] | 69 | 28 | 0 | 0 | 1 | 2 |
| Mean[h] | 62±10 | 32±11 | 0±0 | 2±2 | 1±0.5 | 3±2 |

[f] Percent metalation for all replicates reflect averages from 2-3 fractions as shown in Supplementary Table 5a.
[g] Fig. 5a
[h] Percent metalation with each metal was calculated from the ratio of [metal]/[total metal]. Total metal concentration is the sum of all metal concentrations obtained in the trial (*n* = 3 biological replicates ± SD).

**Supplementary Table 6:** Metal contents and percentage occupancies of *in vivo* metalated MncA containing fractions isolated from *E. coli* in 800 μM zinc [a-b].

**a.**

| Replicate and (fraction) | [Mn] (μM) | [Fe] (μM) | [Co] (μM) | [Ni] (μM) | [Zn] (μM) | [Cu] (μM) |
|---|---|---|---|---|---|---|
| 1(19)[d] | 0.84 | 5.69 | 0.00 | 0[c] | 1.35 | 0.29 |
| 2(24)[e] | 0.37 | 2.14 | 0.00 | 0[c] | 0.44 | 0.04 |
| 3(20)[f] | 0.83 | 5.46 | 0.00 | 0.03 | 1.16 | 0.47 |

[a] Total metal determined by ICP-MS from analysis of fractions eluting from SEC (TSK SW3000) (Supplementary Fig. 11 for corresponding SDS-PAGE analyses). *n* = 3 independent biological replicates are shown.
[b] A single fraction of the MncA peak was selected to minimise interference from a partly co-migrating $Zn^{II}$ protein evident in later fractions.
[c] Where ICP-MS returned negative values (<0.06) these have been reported as zero.
[d] Supplementary Fig. 11a
[e] Supplementary Fig. 11b
[f] Supplementary Fig. 11c

**b.**

| Replicate | Mn | Fe | Co | Ni | Zn | Cu |
|---|---|---|---|---|---|---|
| 1[g] | 10 | 70 | 0 | 0 | 17 | 4 |
| 2 | 13 | 73 | 0 | 0 | 15 | 1 |
| 3 | 10 | 69 | 0 | 0 | 15 | 6 |
| Mean[h] | 11±3 | 71±2 | 0±0 | 0±0 | 15±1 | 4±2 |

[g] Supplementary Fig. 11d
[h] Percent metalation with each metal was calculated from the ratio of [metal]/[total metal]. Total metal concentration is the sum of all metal concentrations obtained in the trial (*n* = 3 biological replicates ± SD).

**Supplementary Table 7:** Metal contents and percentage occupancies of *in vivo* metalated MncA containing fractions isolated from *E. coli* in 600 μM copper [a-b].

**a.**

| **Replicate and (fraction)** | **[Mn] (μM)** | **[Fe] (μM)** | **[Co] (μM)** | **[Ni] (μM)** | **[Zn] (μM)** | **[Cu] (μM)** |
|---|---|---|---|---|---|---|
| 1A(21)[d,e] | 0.65 | 6.77 | 0.00 | 0[c] | 0.11 | 2.45 |
| 1B(21)[d] | 0.23 | 2.55 | 0.00 | 0[c] | 0.13 | 0.25 |
| 2(21)[f] | 0.77 | 7.19 | 0.00 | 0[c] | 0.34 | 0.04 |
| 3(24)[g] | 0.60 | 4.60 | 0.00 | 0[c] | 0.19 | 0.11 |

[a] Total metal determined by ICP-MS from analysis of fractions eluting from SEC (TSK SW3000) (Supplementary Fig. 12-13 for corresponding SDS-PAGE analyses). *n* = 3 independent biological replicates are shown.
[b] A single fraction of the MncA peak was selected to minimise interference from a partly co-migrating $Cu^{I}$ protein tentatively GAPDH.
[c] Where ICP-MS returned negative values (<0.12) these have been reported as zero.
[d] Replicate 1A and 1B originate from a common biological extract, except 1B was the second largest fraction from the SEC further processed through a Blue Sepharose column to remove putative GAPDH. Only the data from 1B was propagated along with replicates 2 and 3.
[e] Supplementary Fig. 12
[f] Supplementary Fig. 13a
[g] Supplementary Fig. 13b

**b.**

| **Replicate** | **Mn** | **Fe** | **Co** | **Ni** | **Zn** | **Cu** |
|---|---|---|---|---|---|---|
| 1A | 7 | 68 | 0 | 0 | 1 | 25 |
| 1B | 7 | 83 | 0 | 0 | 4 | 8 |
| 2[h] | 9 | 87 | 0 | 0 | 4 | 2 |
| 3 | 11 | 85 | 0 | 0 | 3 | 1 |
| Mean[i,j] | 9±4 | 85±4 | 0±0 | 0±0 | 4±1 | 4±4 |

[h] Supplementary Fig. 13b
[i] Replicate 1A, not purified with Blue Sepharose, was excluded from the calculated occupancy (*n* = 3 biological replicates ± SD).
[j] Percent metalation with each metal was calculated from the ratio of [metal]/[total metal]. Total metal concentration is the sum of all metal concentrations obtained in the trial.

**Supplementary Table 8:** Metal contents and percentage occupancies of *in vivo* metalated MncA containing fractions isolated from *E. coli* in 600 μM nickel [a-b].

**a.**

| Replicate and (fraction) | [Mn] (μM) | [Fe] (μM) | [Co] (μM) | [Ni] (μM) | [Zn] (μM) | [Cu] (μM) |
|---|---|---|---|---|---|---|
| 1(19)[c] | 0.15 | 2.65 | 0.01 | 14.26 | 0.40 | 0.48 |
| 2(21)[d] | 0.18 | 6.42 | 0.03 | 14.97 | 0.12 | 0.04 |
| 3(22)[e] | 0.45 | 7.31 | 0.01 | 25.69 | 0.32 | 0.26 |

[a] Total metal determined by ICP-MS from analysis of fractions eluting from SEC (TSK SW3000) (Supplementary Fig. 14 for corresponding SDS-PAGE analyses). *n* = 3 independent biological replicates are shown.

[b] A single fraction of the MncA peak was selected to minimise interference from a partly co-migrating zinc and/or copper protein(s) evident in earlier replicates (Supplementary Tables 6-7).

[c] Supplementary Fig. 14a

[d] Supplementary Fig. 14b

[e] Supplementary Fig. 14c

**b.**

| Replicate | Mn | Fe | Co | Ni | Zn | Cu |
|---|---|---|---|---|---|---|
| 1 | 1 | 15 | 0 | 79 | 2 | 3 |
| 2[f] | 1 | 29 | 0 | 69 | 1 | 0 |
| 3 | 1 | 21 | 0 | 75 | 1 | 1 |
| Mean[g] | 1±1 | 22±7 | 0±0 | 75±5 | 1±1 | 1±1 |

[f] Supplementary Fig. 14d

[g] Percent metalation with each metal was calculated from the ratio of [metal]/[total metal]. Total metal concentration is the sum of all metal concentrations obtained in the trial (*n* = 3 biological replicates ± SD).

**Supplementary Table 9:** Metal contents and percentage occupancies of *in vivo* metalated MncA containing fractions isolated from *E. coli* in 300 μM cobalt [a].

**a.**

| Replicate and (fraction) | [Mn] (μM) | [Fe] (μM) | [Co] (μM) | [Ni] (μM) | [Zn] (μM) | [Cu] (μM) |
|---|---|---|---|---|---|---|
| 1(20)[b] | 1.20 | 0.47 | 7.13 | 0.05 | 0.00 | 0.08 |
| 1(21)[b] | 1.20 | 0.49 | 7.27 | 0.06 | 0.01 | 0.05 |
| 1(22)[b] | 0.49 | 0.25 | 2.81 | 0.07 | 0.33 | 0.04 |
| 2(17)[c] | 1.45 | 1.16 | 5.73 | 0.00 | 0.19 | 0.08 |
| 2(18)[c] | 2.17 | 1.71 | 8.64 | 0.00 | 0.21 | 0.03 |
| 2(19)[c] | 0.89 | 0.73 | 3.55 | 0.00 | 0.02 | 0.02 |
| 3(9)[d] | 1.31 | 0.97 | 6.34 | 0.06 | 0.14 | 0.26 |
| 3(10)[d] | 0.40 | 0.26 | 1.91 | 0.11 | 0.35 | 0.08 |

[a] Total metal determined by ICP-MS from analysis of fractions eluting from SEC (TSK SW3000) (Supplementary Fig. 15 for corresponding SDS-PAGE analyses). *n* = 3 independent biological replicates are shown.
[b] Supplementary Fig. 15a
[c] Supplementary Fig. 15b
[d] Supplementary Fig. 15c

**b.**

| Replicate | Mn | Fe | Co | Ni | Zn | Cu |
|---|---|---|---|---|---|---|
| 1 | 13 | 6 | 77 | 1 | 3 | 1 |
| 2[e] | 17 | 14 | 68 | 0 | 1 | 0 |
| 3 | 14 | 9 | 66 | 2 | 6 | 3 |
| Mean[f] | 15±2 | 10±4 | 70±6 | 1±1 | 4±3 | 1±1 |

[e] Supplementary Fig. 15d
[f] Percent metalation with each metal was calculated from the ratio of [metal]/[total metal]. Total metal concentration is the sum of all metal concentrations obtained in the trial (*n* = 3 biological replicates ± SD).

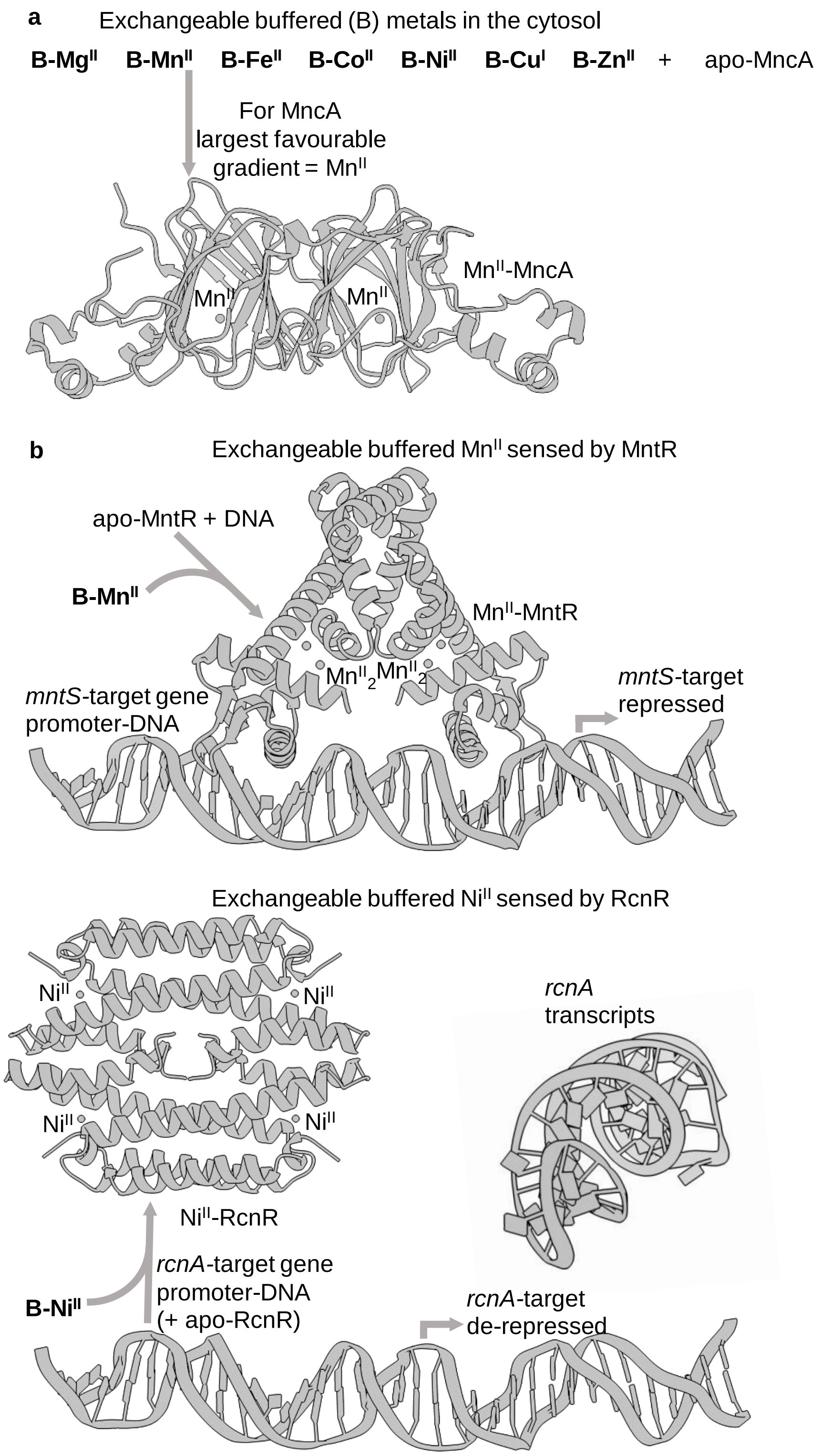


**Supplementary Fig. 1. A model for the speciation of metalation**. **a** It is thought that the speciation of metalation depends on the preferences of a protein for different metals competing at intracellular metal availabilities. Cells contain pools of available metals that can exchange from buffering molecules (B) to tighter ligands including metalloproteins. The most competitive metal is the one where the gradient in free energy of complex formation, from buffering molecules to the protein, is most favourable (the largest $-\Delta\Delta G$). In the native host this must be $Mn^{II}$ for $Mn^{II}$-MncA (PDB 2VQA)[2]. Calculating these gradients requires

knowledge of how tightly each metal is bound to its pool of buffer molecules and how tightly it binds to the protein. The latter can generally be determined from metal affinities ($K_A$) of the protein. This is converted to a free energy for complex formation ($\Delta G_{MP}$) via the standard relationship $\Delta G = -RT\ln K_A$ ($R$ = gas constant, $T$ = temperature). MncA kinetically traps metals so affinities can't be directly measured and here we use an alternative approach to determine relative binding preferences. **b** DNA-binding metal sensors can be used to estimate how tightly exchangeable metals are bound to the pool of buffering molecules. For example, intracellular available $Mn^{II}$ is detected by MntR (PDB 9C4D)[3]. DNA binding by MntR, and likewise sensors for other metals, allosterically switches on metal-binding. This in turn regulates transcription of known metal-responsive genes. Thus, the abundance of transcripts encoded by a metal regulated gene reports on metal binding to the sensor protein. Provided the relationship between available [metal] and transcript abundance has been calibrated then qPCR can provide a read-out of available [metal]: This can be expressed as a free energy of complex formation to a buffer ligand that would be 50% saturated at the [metal] ($\Delta G_M$). $Mn^{II}$-MntR, and most other metal sensors in *E. coli* and *Salmonella*, have already been calibrated, but $Ni^{II}$-RcnR (modelled using AlphaFold 3, sequences provided in Source data file)[4], had not and is calibrated here. The most favourable gradient, the largest $-\Delta\Delta G$, is calculated from $\Delta G_{MP} - \Delta G_M$. The metalation model is introduced in reviews[5,6].

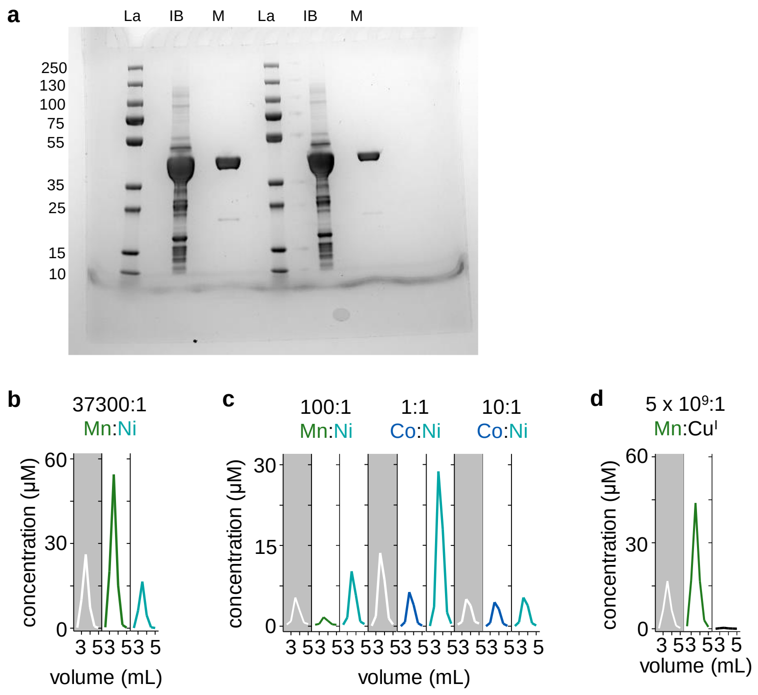


**Supplementary Fig. 2. Refolding MncA to establish relative preference for Ni$^{II}$ and competition with BCA-buffered Cu$^{I}$. a** Full gel image shown in Fig. 1b (representative of >3 analogous purifications of urea solubilised MncA in these studies). Inclusion bodies containing MncA were redissolved in 8 M urea then refolded in urea-free buffer containing competing metal ions followed by concentration via application to an anion exchange column (1 mL Q-Sepharose) and elution with buffer containing 500 mM NaCl. Protein was then visualised with SDS-PAGE (La, protein size markers; IB, dissolved inclusion bodies; M, MncA refolded with 10 µM Ni$^{II}$, the three lanes were repeated, the second MncA fraction diluted for improved visualisation). **b** Additional competition for metalation of MncA between Ni$^{II}$ and Mn$^{II}$ in a histidine metal buffer used along with trials 1-3 (Fig. 1c) to calculate the preference for Ni$^{II}$ relative to Mn$^{II}$ ($n$ = 4 experimental replicates ± SD). **c** The relative preference for Ni$^{II}$ was also investigated by competing Ni$^{II}$ against Mn$^{II}$ and against Co$^{II}$ in the absence of a metal buffer (Supplementary Table 2). $n$ = 3 independent replicates shown. **d** MncA is predominantly metalated with Mn$^{II}$ in competition with BCA-buffered Cu$^{I}$. MncA was folded in 50 mM MOPS, pH 7.5, buffer containing 15 µM $MnCl_2$ and 50 µM CuCl buffered by 398 µM BCA (total concentration). Hydroxylamine (1 mM) was added to maintain Cu$^{I}$ in a reduced state. Mn$^{II}$ is not buffered by BCA, while Cu$^{I}$ is buffered to $3 \times 10^{-15}$ M under these conditions[7], creating a Mn$^{II}$ to Cu$^{I}$ ratio of $5 \times 10^{9}$. MncA recovered from this solution (as in Fig. 1c) contained 99.3% Mn$^{II}$ and 0.7% Cu$^{I}$, as determined by ICP-MS ($n$ = 1). The preference of MncA for Cu$^{I}$ over Mn$^{II}$ is thus estimated to be less than $4 \times 10^{7}$ consistent with the determined value of $3.97 \times 10^{4}$ (Table 1, Fig. 1c-d). Source data are provided as a Source Data file.

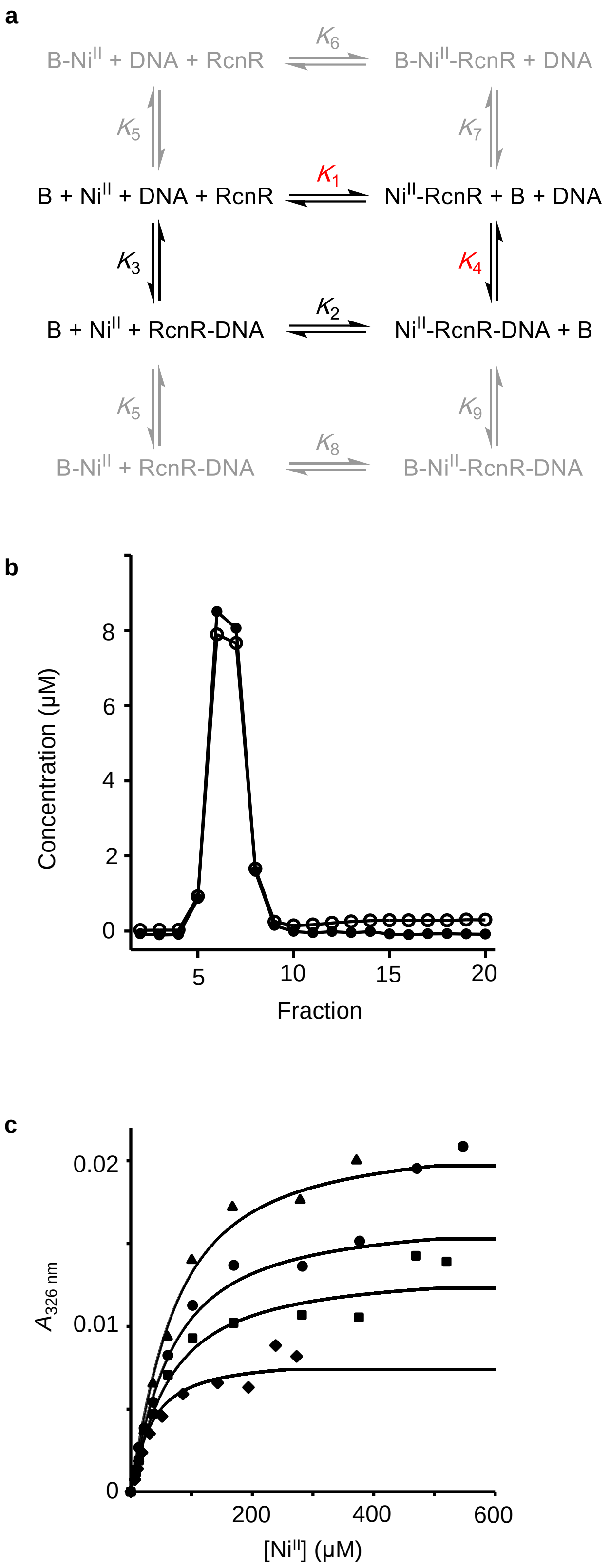


**Supplementary Fig. 3. Characterisation of $Ni^{II}$-RcnR. a** Schematic representation of RcnR in four thermodynamically coupled allosteric states, analogous to other DNA-binding metal-sensing transcriptional regulators[8-11]. RcnR $Ni^{II}$ affinity (as $K_A = K_1$ ($M^{-1}$), or $K_D = 1/K_1$ (M)) and $Ni^{II}$-RcnR affinity for DNA (as $K_4$ or $1/K_4$) were needed (shown red) to calibrate the

relationship between DNA-occupancy ($\theta_D$) and intracellular $Ni^{II}$ availability. Other values to relate DNA-occupancy ($\theta_D$) and intracellular $Co^{II}$ availability are known[8]. Buffered metal (B-$Ni^{II}$) can exchange to and from RcnR via association of buffer (B) and protein molecules (grey wings): Analogous exchange is anticipated to metalate MncA (Supplementary Figure 1). Associative ligand exchange bypasses slow dissociation of metal to a hydrated state. Metalation is then a function of relative strengths of metal binding to proteins of interest (metal-sensor or MncA) versus exchangeable buffer. **b** Confirmation of $Ni^{II}$ binding stoichiometry of RcnR. Co-elution of $Ni^{II}$ and RcnR in protein containing fractions by size exclusion chromatography (20 μM RcnR monomer incubated with 30 μM $Ni^{II}$). RcnR concentration (closed circles) determined by Bradford assay and $Ni^{II}$ concentration (open circles) determined by ICP-MS ($n$ = 1). **c** Titration with $Ni^{II}$ in the presence of EGTA as in Figure 2a using four RcnR monomer concentrations; 40.4 μM RcnR (triangle) and 464 μM EGTA; 31.5 μM RcnR (circle) and 471 μM EGTA; 25.3 μM RcnR (square) and 479 μM EGTA; 15.3 μM RcnR (diamond) and 243 μM EGTA ($n$ = 4 independent experimental replicates). The plotted data corresponds to the feature of $Ni^{II}$-RcnR at absorbance 326 nm. Solid lines represent a simultaneous best fit to all data sets, also illustrated in Figure 2b, giving single site affinity (± SE) 1.18 (± 0.07) x $10^{-12}$ per tetramer equating to 2.36 (± 0.13) x $10^{-12}$ for two allosterically activating sites (fitting model in Supplementary Software). Source data are provided as a Source Data file.

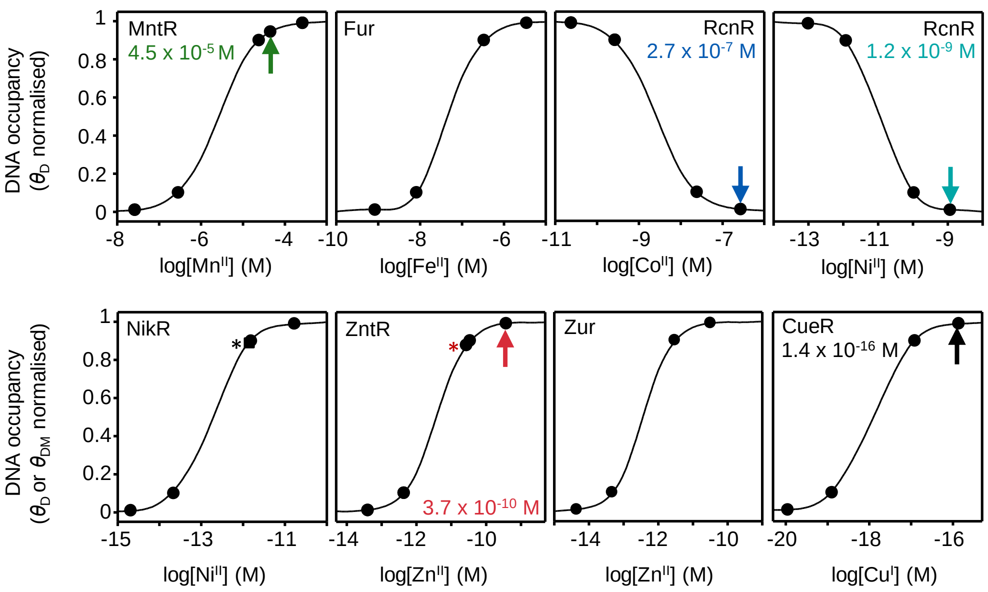


**Supplementary Fig. 4. Calibrated metal sensor responses as a function of intracellular metal availability.** The calculated relationship between intracellular metal availability and normalised DNA occupancy ($\theta_D$) for repressors, or normalised DNA occupancy with metalated sensor ($\theta_{DM}$) for activators, calculated using known affinity and abundance values for each sensor[8], as in Figure 2d for the $Ni^{II}$ responses of RcnR (included here for completeness) using bespoke calculations for metal-dependent activators and co-repressors as in Supplementary Data 2 for metal-dependent de-repressors[8]. The dynamic ranges of each sensor have been defined by $\theta_D$ or $\theta_{DM}$ of 0.01 to 0.99 (Fig. 4c, 5b, 6b, Supplementary Fig. 16, 19, 20) or in Figure 2e and Supplementary Figure 5 by 0.1 to 0.9 values (black circles). Arrows indicate estimates of maximum intracellular metal availabilities in cells cultured in media containing high metal levels (used in Fig. 5b, 6; $Mn^{II}$ 4.5 x $10^{-5}$ M, $Cu^{I}$ 1.4 x $10^{-16}$ M, $Zn^{II}$ 3.7 x $10^{-10}$ M, $Ni^{II}$ 1.2 x $10^{-9}$ M and $Co^{II}$ 2.7 x $10^{-7}$ M): Note that for MntR the values in cells exposed to elevated metal alone depart from the boundary ($\theta_D$ 0.99) obtained in cells exposed to $Mn^{II}$ and $H_2O_2$[12]. For ZntR only the qPCR boundary ($\theta_{DM}$ 0.99 for a metal-dependent activator) was not exceeded here (Fig. 7) and a second data point (red asterisk) approximates availability albeit the boundary value (red arrow) was used in calculations and predictions. Metal availabilities at defined $\theta_D$, and *vice-versa*, were read out with MATLAB code (Supplementary Note 3 available in Osman and coworkers Supplementary Data[8]). The mid-point of the combined range for $Ni^{II}$ from NikR and RcnR is indicated (black asterisk, square) analogous to the arrow and square on Figure 2d for $Ni^{II}$-RcnR. Source data are provided as a Source Data file. qPCR primers in Supplementary Data 9[12-14]

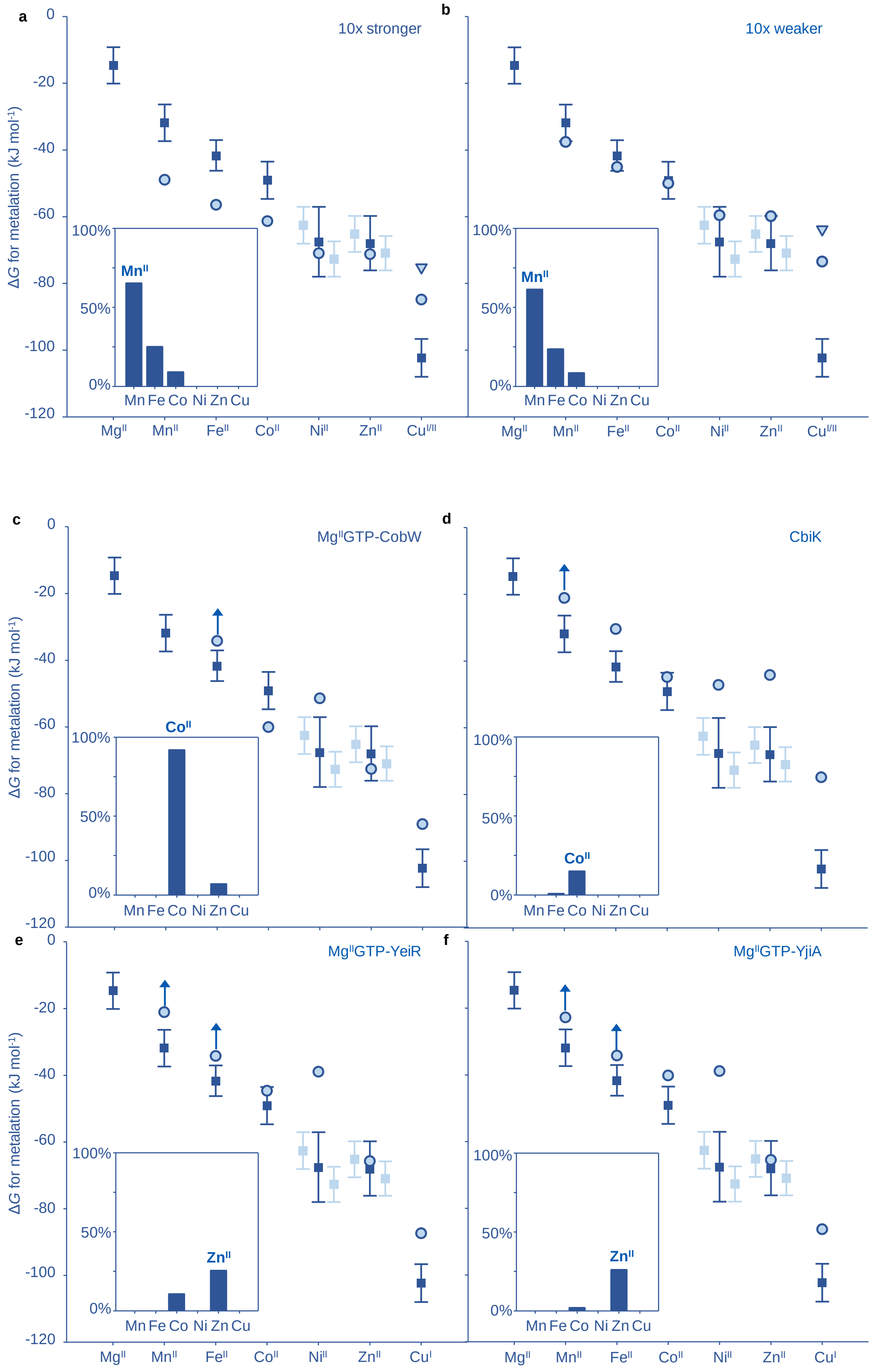


**Supplementary Fig. 5. Relative metal preferences decode metal speciation of MncA and refined mid-range availabilities decode cognate metals of five proteins. a** Assigned

free energy for formation of $Mn^{II}$-MncA decreased by 5.7 kJ $mol^{-1}$ relative to Figure 2e. Other values were calculated relative to $Mn^{II}$-MncA. **b** Assigned free energy for formation of $Mn^{II}$-MncA increased by 5.7 kJ $mol^{-1}$ and other values again calculated relative to $Mn^{II}$-MncA. Inset panels show speciation of metalated MncA in an ideal cell using the $Ni^{II}$-RcnR refined availabilities in Figure 1d and Supplementary Data 3. In both cases, the metalated fraction of MncA contains 65% $Mn^{II}$, 25% $Fe^{II}$ and 9% $Co^{II}$, identical to the values shown in Figure 2e inset. Thus, the speciation of metalation does not vary with absolute free energies for complex formation but is a function of the relative preferences for different metals. However, when 5.7 kJ $mol^{-1}$ lower in (**a**) total metalation is 99.9% but 93.4% when 5.7 kJ $mol^{-1}$ higher in (**b**), and 99.4% as in Figure 2e. **c-f** $Ni^{II}$-RcnR-revised idealised blueprint correctly decodes (using Supplementary Data 3) metals of four exemplar proteins, as in insets: CobW, a $Co^{II}$ metallochaperone from *Rhodobacter* (**c**) predicted 92% metalated with $Co^{II}$ in $Mg^{II}$GTP-form. CbiK, a $Co^{II}$ chelatase from *Salmonella* (**d**), predicted predominantly metalated with $Co^{II}$ using known affinities[8]. Greater $Co^{II}$ metalation occurs with reported $Co^{II}$ $K_m$ CbiK[15]. YeiR, a putative $Zn^{II}$ chaperone from *Salmonella* (**e**), predicted predominantly $Zn^{II}$-metalated using affinities of $Mg^{II}$GTP-form. YjiA, a putative $Zn^{II}$ chaperone[16] (**f**), predominantly metalated with $Zn^{II}$ in $Mg^{II}$GTP-form. Predictions in (**c-f**) excluded limiting affinities (noted with arrows) where protein was unable to compete with dye in the assay. The weakest limit to the $Mn^{II}$ affinity of CbiK has been adjusted to 2 x $10^{-4}$, consistent with <10% metalation by $Mn^{II}$ observed in[8]. Cognate metals are correctly decoded as $Mn^{II}$ for MncA (**a**, **b**), $Co^{II}$ for CobW (**c**), $Co^{II}$ for CbiK (**d**), $Zn^{II}$ for YeiR (**e**) and $Zn^{II}$ for YjiA (**f**). In all panels bars are sensor ranges, 10% to 90%, $\Delta G_M$ squares, $\Delta G_{MP}$ pale blue circles ($Cu^I$ triangle in **a**, **b**). Source data are provided as a Source Data file.

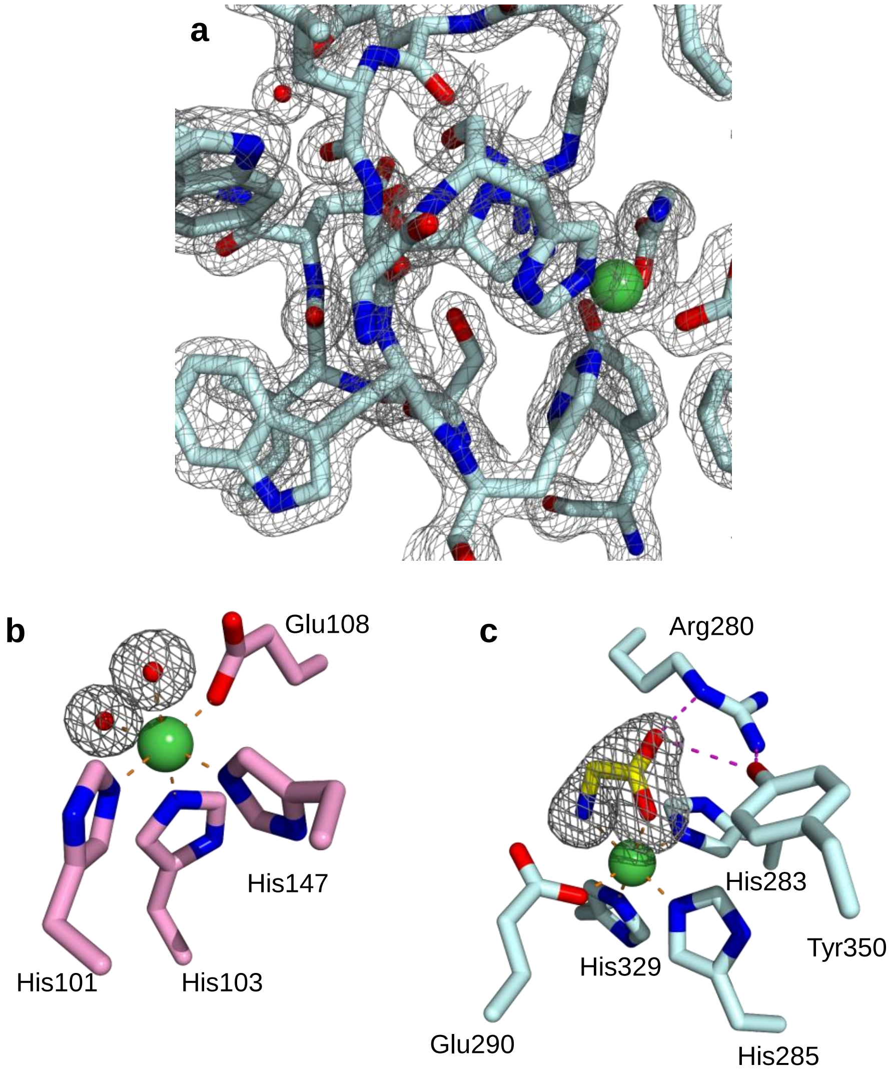


**Supplementary Fig. 6. Electron density maps for (Ni$^{II}$)$_2$MncA. a** Sample of the 2$F_o$-$F_c$ electron density map, contoured at 2 $\sigma$, obtained from refinement of the (Ni$^{II}$)$_2$MncA structure to 1.60 Å resolution with $R_{work}$/$R_{free}$ of 16.3%/17.6% (Supplementary Table 3). Overall, the model includes residues 39-394 for chains A and B, and 39-391 for chain C. Ni$^{II}$ ions were modelled into the known Mn$^{II}$ sites in the two cupin domains[2]. Ni$^{II}$ is coordinated in a similar environment to that of Mn$^{II}$ in (Mn$^{II}$)$_2$MncA[2]. Here, the map is shown covering the region adjacent to the C-terminal metal binding site (light blue carbons). **b, c** Omit electron density maps (contoured to 16 $\sigma$) generated by removing non-protein ligands from the two MncA-bound Ni$^{II}$ ions. **b** Binding of Ni$^{II}$ to three histidine imidazole nitrogen atoms and one glutamate carboxyl oxygen is visualised along with two solvent molecules completing the coordination sphere in the amino-terminal site (pink carbons). **c** A small molecule solute is present in the carboxy-terminal site (blue carbons), indicated by electron density adjacent to the Ni$^{II}$ ion. Stereochemistry, which includes likely hydrogen-bonding to Arg280 and Tyr350 adjacent to the site, and unresolved density in $F_o$-$F_c$ maps, suggest that the ligand is glycine, probably originating from solubilised inclusion bodies used to prepare Ni$^{II}$-metalated MncA, which was folded without a metal buffer. Other possible ligands, including acetate and carbonate, with and without additional solvent ligands, did not fit the density or meet the H-bonding and/or metal-bonding requirements of the site. Data collected using crystals obtained from a separate preparation of (Ni$^{II}$)$_2$MncA also indicated Ni$^{II}$-bound glycine. In the (Mn$^{II}$)$_2$MncA structure (PDB ID 2VQA) acetate was modelled in this location likely originating

from the crystallization solutions[2]. The small molecules in both structures are thought to reflect the substrate position. Metal-ligand bonds are shown as orange dashes. Hydrogen bonds to glycine are shown as purple dashes. In both domains, The figure was made with PyMOL.

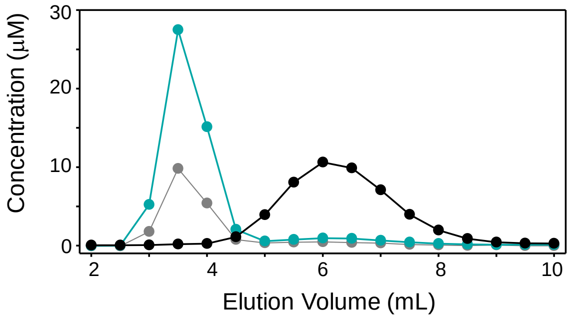


**Supplementary Fig. 7. $Ni^{II}$ is kinetically trapped by MncA.** MncA was refolded in 10 μM nickel sulphate and metalated protein recovered by anion exchange chromatography (Q Sepharose, 50 mM Tris, pH 7.5, eluted with 500 mM NaCl). A solution of $Ni^{II}$-bound protein (22 μM) was incubated with 44 μM cupric sulphate for 24 h, EDTA added to a concentration of 100 μM for 30 minutes, before separation by size exclusion chromatography (PD-10 column) collecting 0.5 mL fractions, which were analysed for metals by ICP-MS and protein ($A_{280nm}$). $Ni^{II}$ (teal line) co-elutes with MncA (grey line), while $Cu^{II}$ (black line) remains dissociated. MncA is calculated to be 99.9% metalated with $Ni^{II}$ even though MncA exhibits a greater preference for $Cu^{II}$ ($1.9 \times 10^6$ relative to $Mn^{II}$) than $Ni^{II}$ ($7.2 \times 10^3$ relative to $Mn^{II}$) at folding (Table 1, Fig. 1d) ($n = 1$). Source data are provided as a Source Data file.

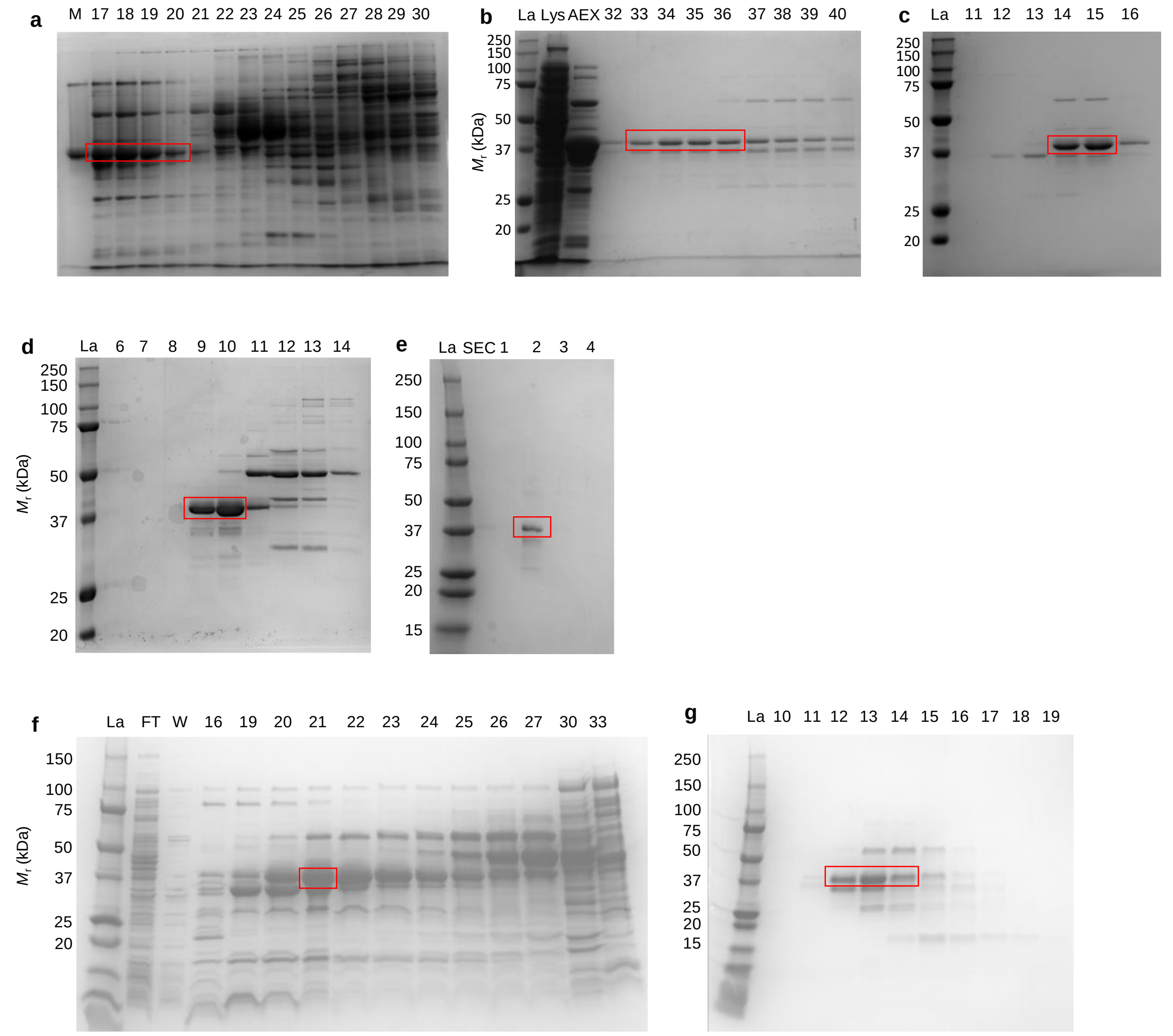


**Supplementary Fig. 8. SDS-PAGE of MncA purification in soluble form following expression in *E. coli*.** Crude lysates were purified by anion exchange (5 mL Q-Sepharose). **a** Full image of fractions analysed by SDS-PAGE (M, marker = purified recombinant MncA, related to commercial markers in other analyses; 17-30 fractions approximately 1 mL each), fractions 17-20 (red box and Fig. 4a gel inset) were pooled and resolved by size exclusion chromatography (Superdex 200). **b** Fractions analysed by SDS-PAGE (La, protein size markers kDa left; Lys, crude lysate; AEX pooled fractions 17-20; 32-40, fractions after size exclusion chromatography approximately 1 mL each) fractions 33-36 (red box) were pooled and resolved by anion exchange chromatography (1 mL Q-Sepharose). **c** Fractions analysed by SDS-PAGE (La, protein size markers kDa left; 11-16 fraction numbers ~1 mL each) fractions 14-15 (red box) were analysed for percentage metal occupancies (Supplementary Table 4). Lysates from two further independent biological replicates purified as above ($n$ = 3). **d** SDS-PAGE following final anion exchange chromatography (La, protein size markers kDa left; 6-14 fraction numbers ~1 mL each, as shown in Fig. 4b). Fractions 9-10 (red box) were analysed for percentage metal occupancies (Supplementary Table 4, Fig. 4b) (this replicate used Superdex 75, not 200, in second chromatography step). **e** SDS-PAGE following final anion exchange chromatography (La, protein size markers kDa left; SEC, pooled fractions prior to anion exchange; 1-4 fraction numbers ~1 mL each) fraction 2 (red box) analysed for percentage metal occupancies (Supplementary Table 4). **f-g** Alternative simplified analytical protocol. Crude lysates purified using anion exchange

chromatography (5 mL Q-Sepharose). Fractions analysed by SDS-PAGE (**f**) (La, protein size markers kDa left; FT, unbound flow through; W, column wash prior to elution with NaCl; 16-33 eluted fractions approximately 1 mL each). Fraction 21 (red box) was resolved by analytical size exclusion HPLC (TSK SW3000). Fractions analysed by SDS-PAGE ($n$ = 1) (**g**) (La, protein size markers kDa left; 10-19 fractions approximately 0.5 mL each). Fractions 12-14 (red box) were analysed for percentage metal occupancies (Supplementary Table 4) ($n$ = 1). Panels **a-e** relate to $n$ = 3 biologically independent replicates in Supplementary Table 4.

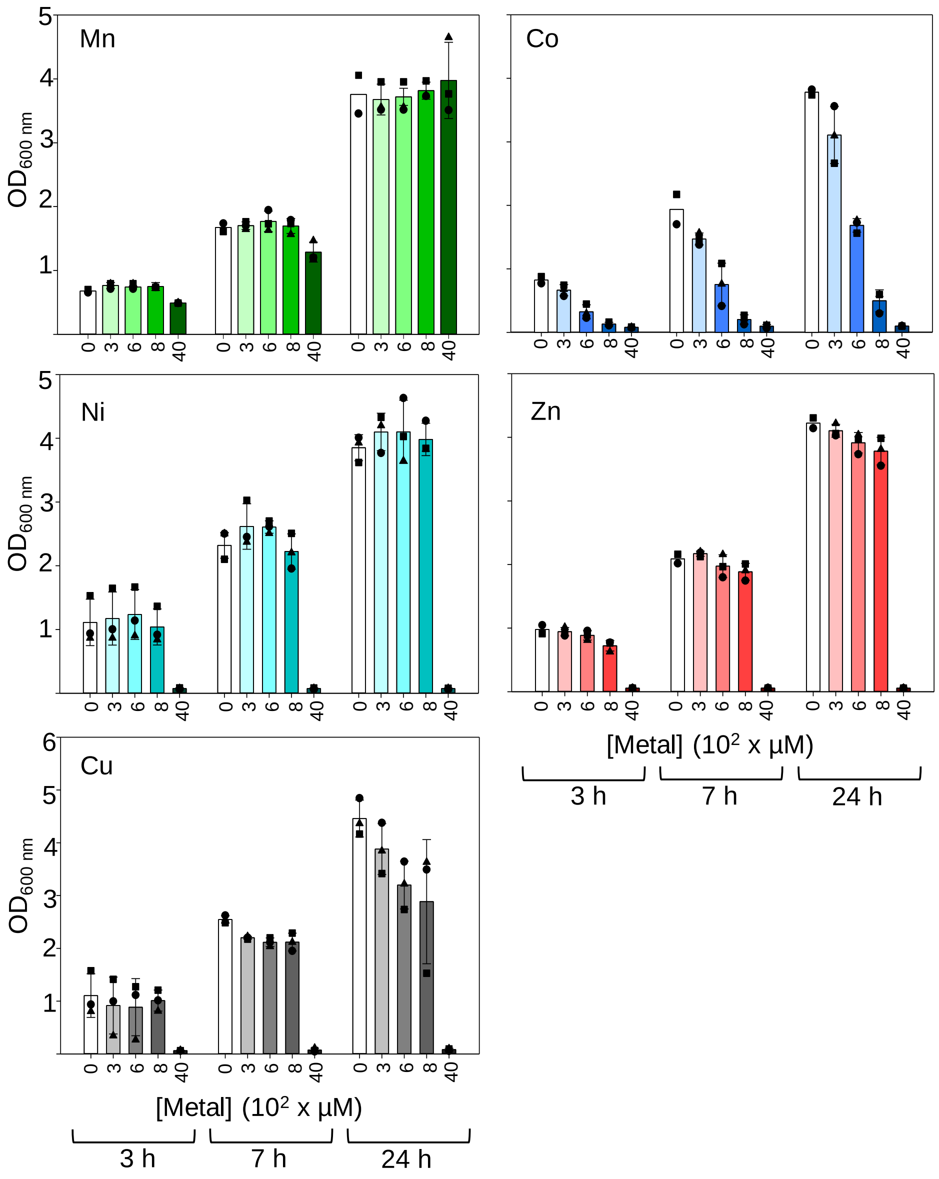


**Supplementary Fig. 9. Effects of elevated metals on growth of *E. coli*.** Growth of *E. coli* at 3, 7 and 24 h after inoculation in LB media. Exposures to manganese (green), cobalt (blue), nickel (teal), zinc (red) and copper (black) at exogenous levels of 0, 300 μM, 600 μM, 800 μM and 4 mM (total metal in the culture). Levels used to maximise intracellular metal availabilities, as indicated by qPCR, were selected to give no, or only modest, inhibition of growth. Hence 4 mM was only selected for manganese and 300 μM was selected for cobalt. $n$ = 3 independent biological replicates (square, circle, triangle) ± SD for all metal treatments. $n$ = 12 independent biological replicates for growth in un-supplemented media composed of $n$ = 3 ± SD in experiments comparing effects of nickel and copper, $n$ = 2 comparing effects of manganese, cobalt and zinc. Source data are provided as a Source Data file.

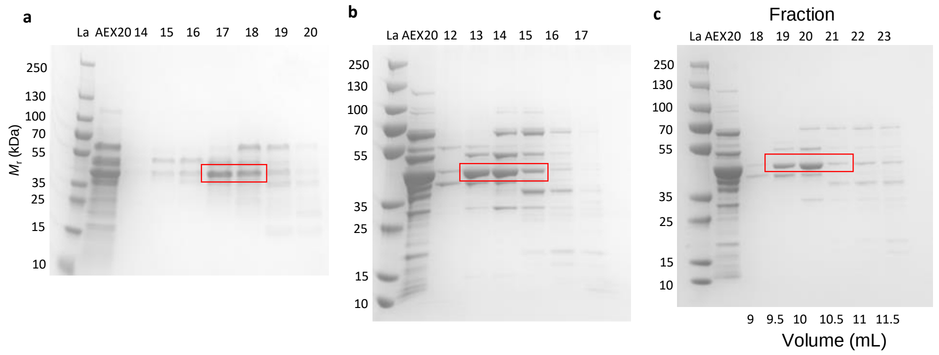


**Supplementary Fig. 10. SDS-PAGE of MncA purified in soluble form following expression in *E. coli* in 4 mM manganese.** Crude lysates were purified using anion exchange chromatography (5 mL Q-Sepharose) followed by size exclusion chromatography (TSK SW3000) and **a-c** SDS-PAGE ($n$ = 3 independent biological replicates). Experiment 1 (La, protein size markers kDa left; AEX, sample after anion exchange; 14-20, fraction numbers approximately 0.5 mL each) (**a**), fractions 17-18 (red box) were analysed for percentage metal occupancy (Supplementary Table 5). Experiment 2 (La, protein size markers kDa left; AEX, sample after anion exchange; 12-17, fraction numbers approximately 0.5 mL each) (**b**), fractions 13-15 (red box) were analysed for percentage metal occupancy (Supplementary Table 5). Experiment 3 (La, protein size markers kDa left; AEX, sample after anion exchange; 18-23, fraction numbers approximately 0.5 mL each) (**c**), fractions 19-21 (red box) were analysed for percentage metal occupancy (Supplementary Table 5, Fig. 5a). Replicated mean percentage occupancies are calculated in Supplementary Table 5.

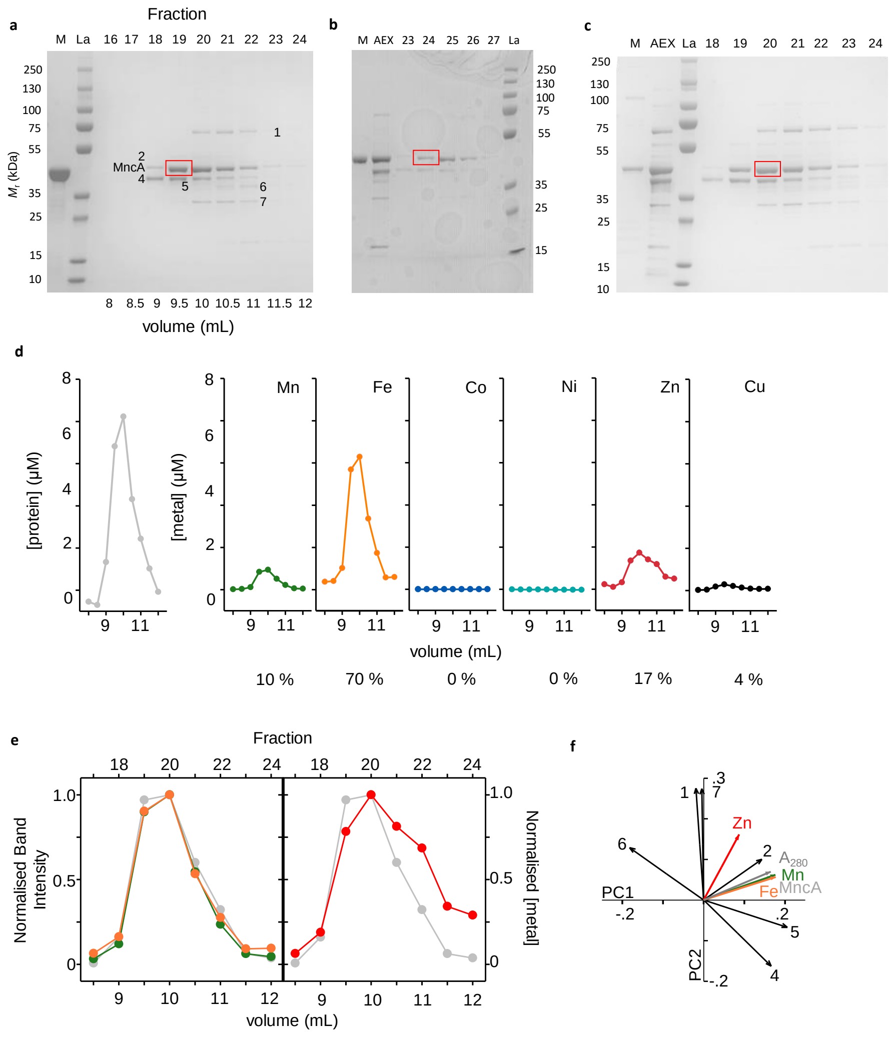

**Supplementary Fig. 11. SDS-PAGE and exemplar chromatogram of MncA purified in soluble form following expression in *E. coli* in 800 µM zinc.** Crude lysates purified using anion exchange chromatography (5 mL Q-Sepharose) followed by size exclusion chromatography (TSK SW3000) and **a-c** SDS-PAGE ($n$ = 3 independent biological replicates). Experiment 1 (M, purified MncA; La, size markers kDa left; 16-24, fractions approximately 0.5 mL each) (**a**), fraction 19 (red box) analysed for percentage metal occupancy (Supplementary Table 6). Experiment 2 (M, purified MncA; AEX, sample after anion exchange; 23-27, fractions approximately 0.5 mL each; La, size markers kDa right) (**b**), fraction 24 (red box) analysed for percentage metal occupancy (Supplementary Table 6). Experiment 3 (M, purified MncA; AEX, sample after anion exchange; La, size markers kDa left; 18-24, fractions approximately 0.5 mL each) (**c**), fraction 20 (red box) analysed for percentage metal occupancy (Supplementary Table 6). **d** Exemplar (experiment 1 of $n$ = 3 independent biological replicates) size exclusion chromatogram showing the MncA containing fractions analysed by ICP-MS. Percentage occupancies from exemplar experiment 1 are shown, while replicated mean percentage occupancies are calculated in Supplementary Table 6 and shown in Figure 6a (top right). **e** Image of SDS-PAGE gel in (**a**) analysed ($n$ = 1) with Image J software to obtain band intensities[17]. Normalised intensity for MncA (grey), corresponding to red boxed band in fraction 19, correlated ($r$ = 1.0) with normalised [$Mn^{II}$] (green) and [$Fe^{II}$] (orange) (left), but imperfectly correlated ($r$ = 0.9) with normalised [$Zn^{II}$] (red) (right). **f** Principal component analysis (*R* software) vectors similarly

cluster for MncA (light grey, obscured), $A_{280nm}$ (dark grey), [$Mn^{II}$] (green), [$Fe^{II}$] (orange), but not for [$Zn^{II}$] which also fails to cluster with any other protein band (numbered as in (**a**), with band 3 labelled as MncA and obsured, note .3 is axis labelling). It is likely that $Zn^{II}$ is partly associated with another protein as well as with MncA and hence fraction 19 (9.5 mL) alone, with least evidence of other proteins, was selected to estimate metalation of MncA. Correlation coefficients were calculated via correl() in Excel. Source data are provided as a Source Data file.

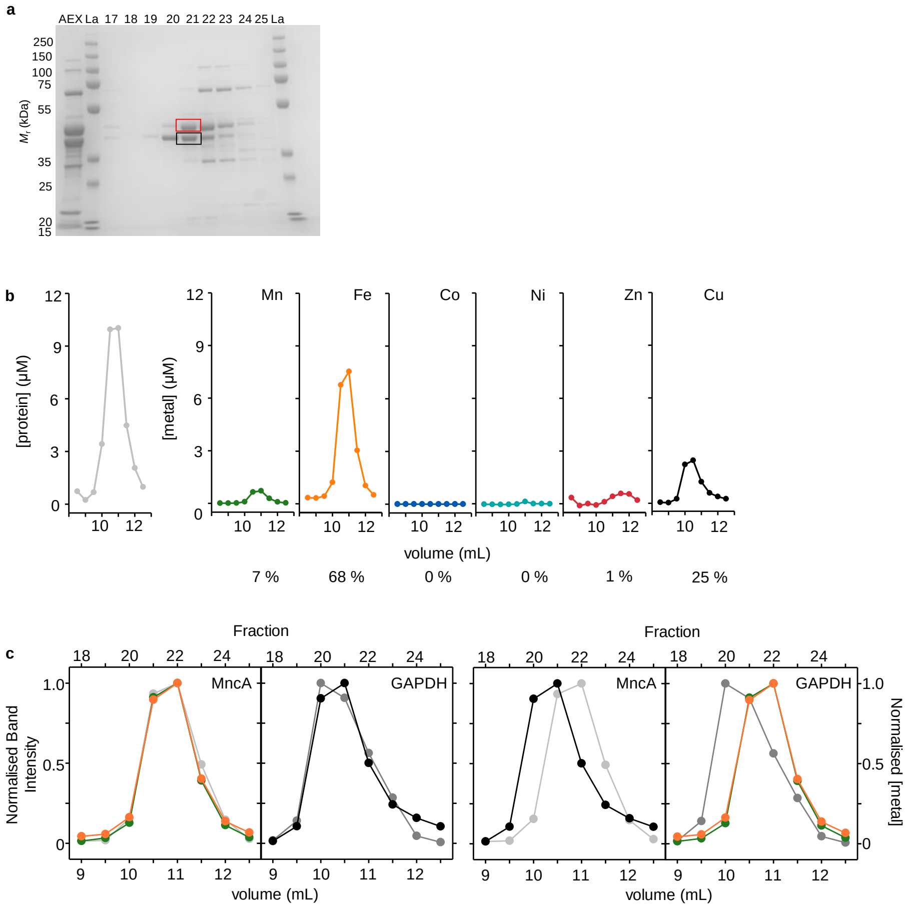


**Supplementary Fig. 12. SDS-PAGE and exemplar chromatogram of MncA purified from *E. coli* in 600 µM copper. a** Crude lysates were purified using anion exchange chromatography (5 mL Q-Sepharose) followed by size exclusion chromatography (TSK SW3000) and SDS-PAGE (AEX, sample after anion exchange; La, protein size markers kDa left; 17-25, fraction numbers approximately 0.5 mL each; La, protein size markers kDa right). Fraction 21 (red box) was analysed but excluded from the mean calculated from Blue Sepharose treated samples (Supplementary Table 7). **b** Size exclusion chromatogram showing the MncA-containing fractions analysed by ICP-MS (fraction 21 corresponds to 10.5 mL). **c** Image of SDS-PAGE gel in (**a**) was analysed by Image J software to obtain band intensities[17]. Normalised intensity (grey) for MncA (red box in (**a**)) and putative GAPDH Mr ~ 36 kDa (black box in (**a**)) correlate with normalised [$Mn^{II}$] (green) and [$Fe^{II}$] (orange) (first left panel), and normalised [copper] (black) (second panel), respectively ($r$ = 1.00 and 0.98). Both align imperfectly with the converse metals (third and fourth panels) ($r$ = 0.60 and 0.58). The $A_{280}$ peak in (**b**) is likely dominated by MncA due to its high extinction coefficient ($\varepsilon$ = 120,000 $M^{-1}$ $cm^{-1}$). Fractions were thus further purified using Blue Sepharose known to bind GAPDH to remove the contaminating copper species (Supplementary Fig. 13)[18]. Correlation coefficients were calculated via correl() in Excel. **a-c**, $n$ = 1. Source data are provided as a Source Data file.

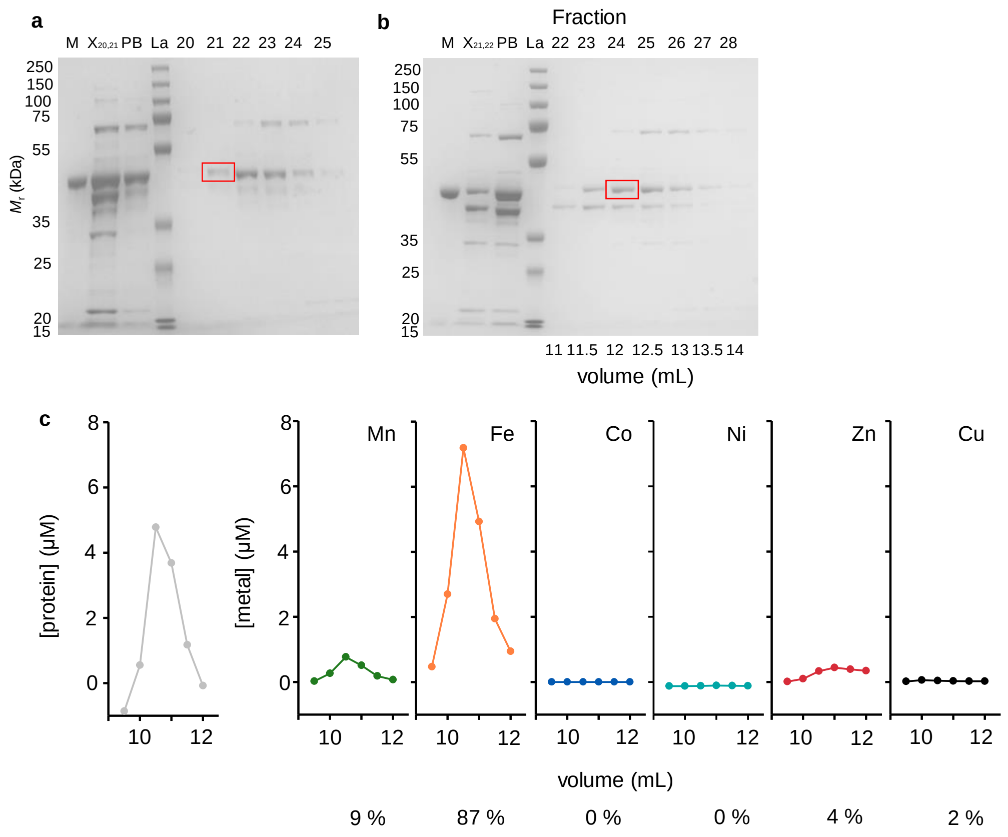


**Supplementary Fig. 13. SDS-PAGE and exemplar chromatogram of MncA further purified from *E. coli* in 600 µM copper with Blue Sepharose.** Crude lysates were purified using anion exchange chromatography (5 mL Q-Sepharose) followed by Blue Sepharose chromatography (5 mL HiTrap Blue HP), size exclusion chromatography (TSK SW3000) and **a, b** SDS-PAGE (*n* = 3 independent biological replicates, one replicate analysed by SDS-PAGE prior to Blue Sepharose treatment in Supplementary Fig. 12a). Experiment 2 (M, purified MncA; X, pooled fractions 20 and 21 following anion exchange prior to Blue Sepharose treatment; PB, fraction eluted following Blue Sepharose purification; La, protein size markers kDa left; 20-25, fraction numbers approximately 0.5 mL each) (**a**) fraction 21 (red box) was analysed for percentage metal occupancy (Supplementary Table 7). Experiment 3 (M, purified MncA; X, pooled fractions 21 and 22 following anion exchange prior to Blue Sepharose treatment; PB, fraction eluted following Blue Sepharose purification; La, protein size markers kDa left; 22-28, fraction numbers approximately 0.5 mL each) (**b**) fraction 24 (red box) was analysed for percentage metal occupancy (Supplementary Table 7). Relative to MncA, putative GAPDH was substantially reduced following treatment with Blue Sepharose (compare with Supplementary Fig. 12a). **c** Exemplar (experiment 2 of *n* = 3 independent biological replicates) size exclusion chromatogram showing the MncA-containing fractions analysed by ICP-MS. Percentage occupancies from exemplar experiment 2 are shown while replicated mean percentages are calculated in Supplementary Table 7 and shown in Figure 6a (top left). Notably, $Fe^{II}$ and $Mn^{II}$ (associated with MncA) are retained, copper (associated with putative GADPH) was largely removed following treatment with Blue Sepharose. Source data are provided as a Source Data file.

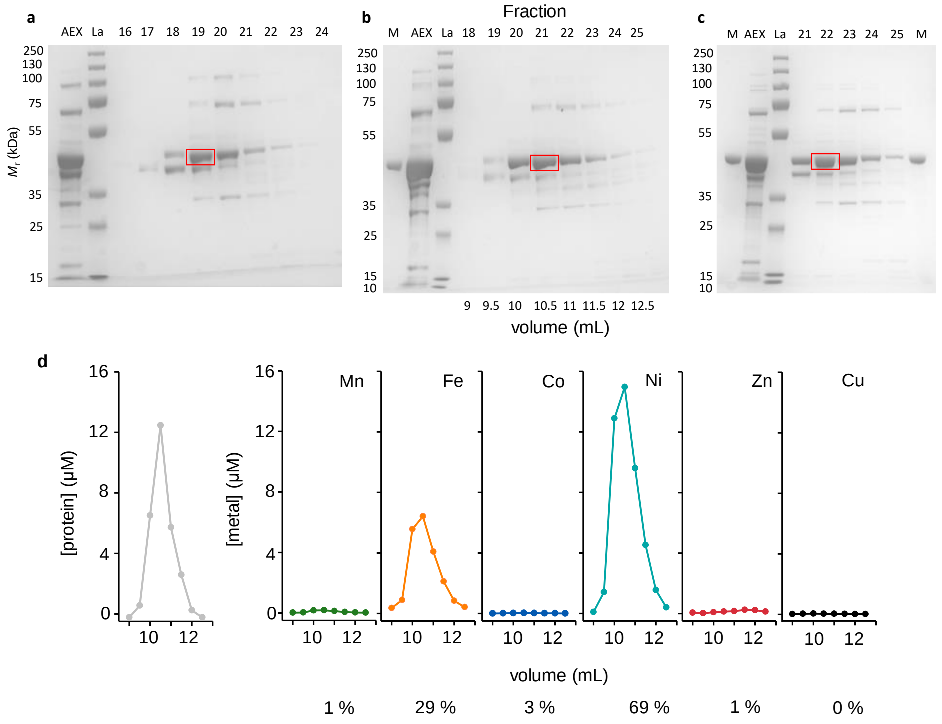


**Supplementary Fig. 14. SDS-PAGE and exemplar chromatogram of MncA purified in soluble form following expression in *E. coli* in 600 µM nickel.** Crude lysates were purified using anion exchange chromatography (5 mL Q-Sepharose) followed by size exclusion chromatography (TSK SW3000) and **a-c** SDS-PAGE (*n* = 3 independent biological replicates). Experiment 1 (AEX, sample after anion exchange; La, protein size markers kDa left; 16-24, fraction numbers approximately 0.5 mL each) (**a**), fraction 19 (red box) was analysed for percentage metal occupancy (Supplementary Table 8). Experiment 2 (M, purified MncA; AEX, sample after anion exchange; La, protein size markers kDa left; 18-25, fraction numbers approximately 0.5 mL each) (**b**), fraction 21 (red box) was analysed for percentage metal occupancy (Supplementary Table 8). Experiment 3 (AEX, sample after anion exchange; La, protein size markers kDa left; 21-25, fraction numbers approximately 0.5 mL each; M, purified MncA) (**c**), fraction 22 (red box) was analysed for percentage metal occupancy (Supplementary Table 8). **d** Exemplar (experiment 2 of *n* = 3 independent biological replicates) size exclusion chromatogram showing the MncA-containing fractions analysed by ICP-MS. Percentage occupancies from exemplar experiment 2 are shown, while replicated mean percentage occupancies are calculated in Supplementary Table 8 and shown in Figure 6a (bottom left). Source data are provided as a Source Data file.

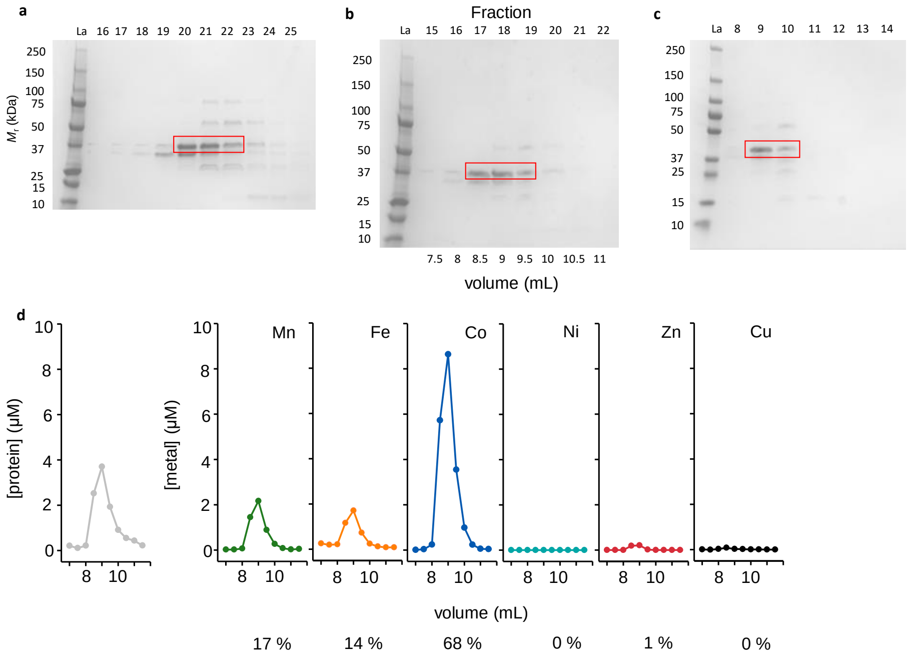


**Supplementary Fig. 15. SDS-PAGE and exemplar chromatogram of MncA purified in soluble form following expression in *E. coli* in 300 μM cobalt.** Crude lysates were purified using anion exchange chromatography (5 mL Q-Sepharose) followed by size exclusion chromatography (TSK SW3000) and **a-c** SDS-PAGE (*n* = 3 independent biological replicates). Experiment 1 (La, protein size markers kDa left; 16-25, fraction numbers approximately 0.5 mL each) (**a**), fractions 20-22 (red box) were analysed for percentage metal occupancy (Supplementary Table 9). Experiment 2 (La, protein size markers kDa left; 15-22, fraction numbers approximately 0.5 mL each) (**b**), fractions 17-19 (red box) were analysed for percentage metal occupancy (Supplementary Table 9). Experiment 3 (La, protein size markers kDa left; 8-14, fraction numbers approximately 0.5 mL each) (**c**), fractions 9-10 (red box) were analysed for percentage metal occupancy (Supplementary Table 9). **d** Exemplar (experiment 2 of *n* = 3 independent biological replicates) size exclusion chromatogram showing the MncA-containing fractions analysed by ICP-MS. Percentage occupancies from exemplar experiment 2 are shown, while replicated mean percentage occupancies are calculated in Supplementary Table 9 and shown in Figure 6a (bottom right). Source data are provided as a Source Data file.

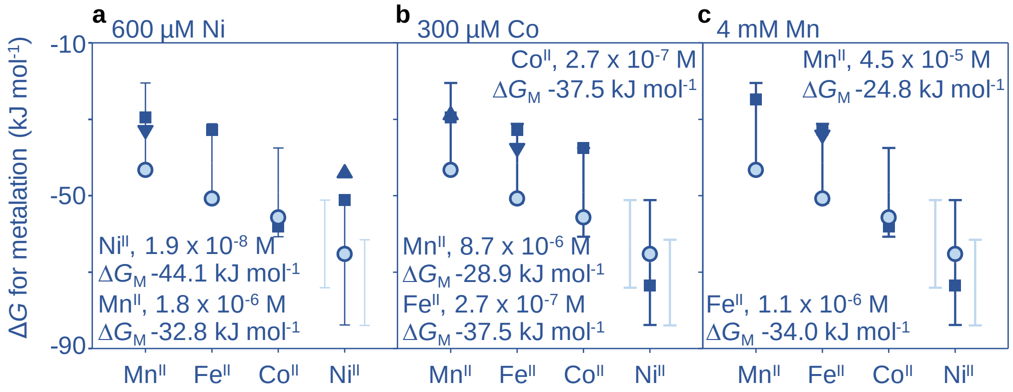


**Supplementary Fig. 16. MncA-refined metal availabilities for *E. coli* in metal-supplemented media.** Medium supplemented with **a** 600 µM nickel, **b** 300 µM cobalt and **c** 4 mM manganese. The preferences of MncA for the respective metals are shown as pale blue circles as in Figure 2e. Intracellular metal availabilities obtained using the calibrated responses of the respective metal sensors and qPCR as in Figures 6b and 5b (dark blue squares). Refined availabilities calculated from the residual differences between observed and predicted occupancies of MncA in Figures 6a, c plus Figure 5b (inset) using Supplementary Data 5 (dark blue triangles), oriented to show direction in which availability was refined. Numerical values of the refined availabilities are shown (bottom left of each panel) along with elevated values for $Co^{II}$ and $Mn^{II}$ in the respective supplemented media (top right of each panel) to serve as blueprints to guide the engineering of metalation of other proteins: Values are included in Supplementary Data 6-8 (along with values for other metals estimated from calibrated responses of metal sensors as in Supplementary Data 4) to predict the metalation states of other proteins in cells cultured in media with added $Ni^{II}$, $Co^{II}$ and $Mn^{II}$. In all panels bars are sensor ranges, 1% to 99%.  Source data are provided as a Source Data file.

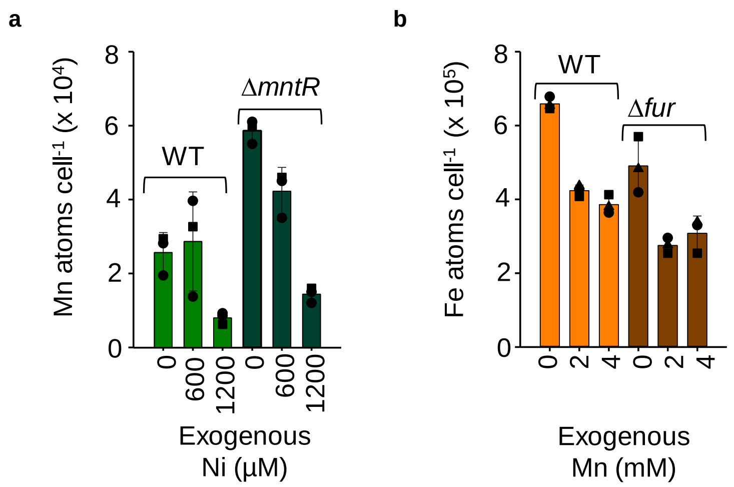


**Supplementary Fig. 17. ICP-MS shows MntR-independent decline in total manganese atoms cell$^{-1}$ in high nickel, and Fur-independent decline in total iron in high manganese. a** Total manganese atoms cell$^{-1}$ decline in high nickel-containing media in both wild type (WT, as in Fig. 8c) and Δ*mntR* with enhanced variation in WT cells exposed to 600 μM nickel. **b** iron atoms cell$^{-1}$ decline in high manganese in both WT and Δ*fur* mutant cells. *n* = 3 independent (square, circle, triangle) biological replicates ± SD. Source data are provided as a Source Data file.

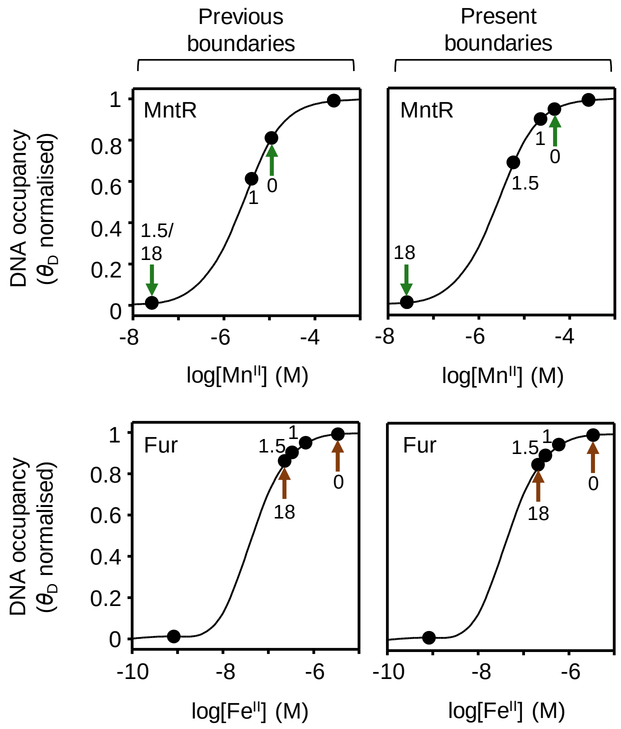


**Supplementary Fig. 18. Shallow Mn$^{II}$ pool depleted after 18 h MncA expression in un-supplemented media.** The qPCR data in Figure 7 was used to make estimations (black circles) of the magnitudes of depletion of labile available Mn$^{II}$ and Fe$^{II}$ during expression of MncA. Calculations were performed by relating transcript abundance at different times to the lower and upper boundaries ($\theta_D$ 0.01 and 0.99) defined using transcripts isolated from cells exposed to EDTA or elevated metal and $H_2O_2$ respectively for metal-dependent co-repressors MntR and Fur as described in[8,12,14]. qPCR values that exceeded the previous boundaries have been assigned the boundary values on the left panels. For comparison the boundaries have been re-assigned to the new extreme qPCR values in the right panels. By either analysis, available Mn$^{II}$ is depleted by 18 h (upper panels) whereas available Fe$^{II}$ shows only modest depletion (lower panels). This is less evident in Figure 7a, b due to the logarithmic nature of qPCR: A single cycle represents 50% of the range in transcript abundance at the highest levels but progressively smaller proportional changes at lower levels of transcript abundance. MncA offers a way to refine estimated metal availabilities where qPCR is less reliable at higher transcript abundance equating to higher metal levels for metal-dependent activators and de-repressors, lower metal levels for metal-dependent co-repressors MntR and Fur: notably metal-responsive transcript abundances are within the more reliable regions of the sensor ranges in *E. coli* grown aerobically in LB media. Source data are provided as a Source Data file.

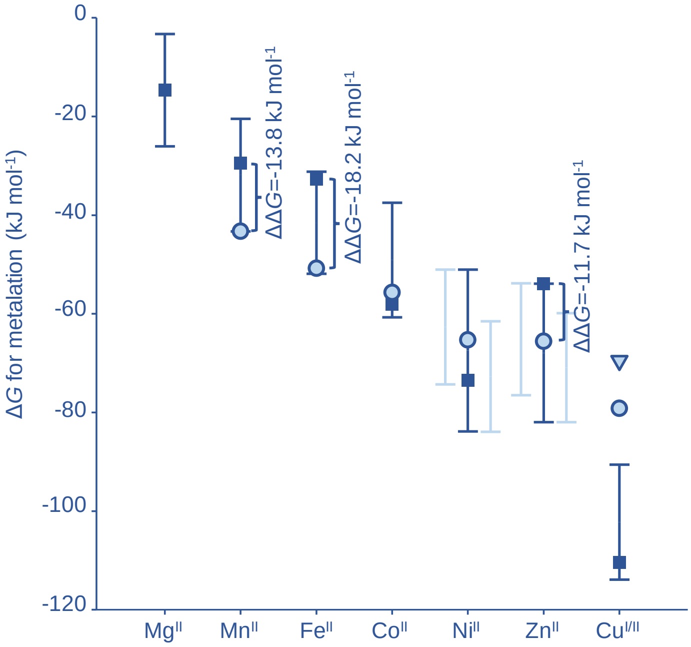


**Supplementary Fig. 19. Larger gradients (ΔΔ*G*) for $Fe^{II}$ and $Mn^{II}$ predicted to inhibit metalation of MncA with $Zn^{II}$ at elevated steady-state $Zn^{II}$ availability.** Symbols are as in Figure 4c and elsewhere showing MncA binding preferences (pale blue circles and triangle for $Cu^{I}$) and exchangeable intracellular metal availabilities (dark blue squares). The largest gradients between availability and MncA binding remain for $Fe^{II}$ and $Mn^{II}$. $Zn^{II}$ shows the third largest gradient. Metalation of MncA, if these availabilities were maintained at steady state, is predicted to be 81% $Fe^{II}$, 14% $Mn^{II}$ and 5% $Zn^{II}$ (Fig. 6a top right). Bars are sensor ranges, 1% to 99%. Source data are provided as a Source Data file.

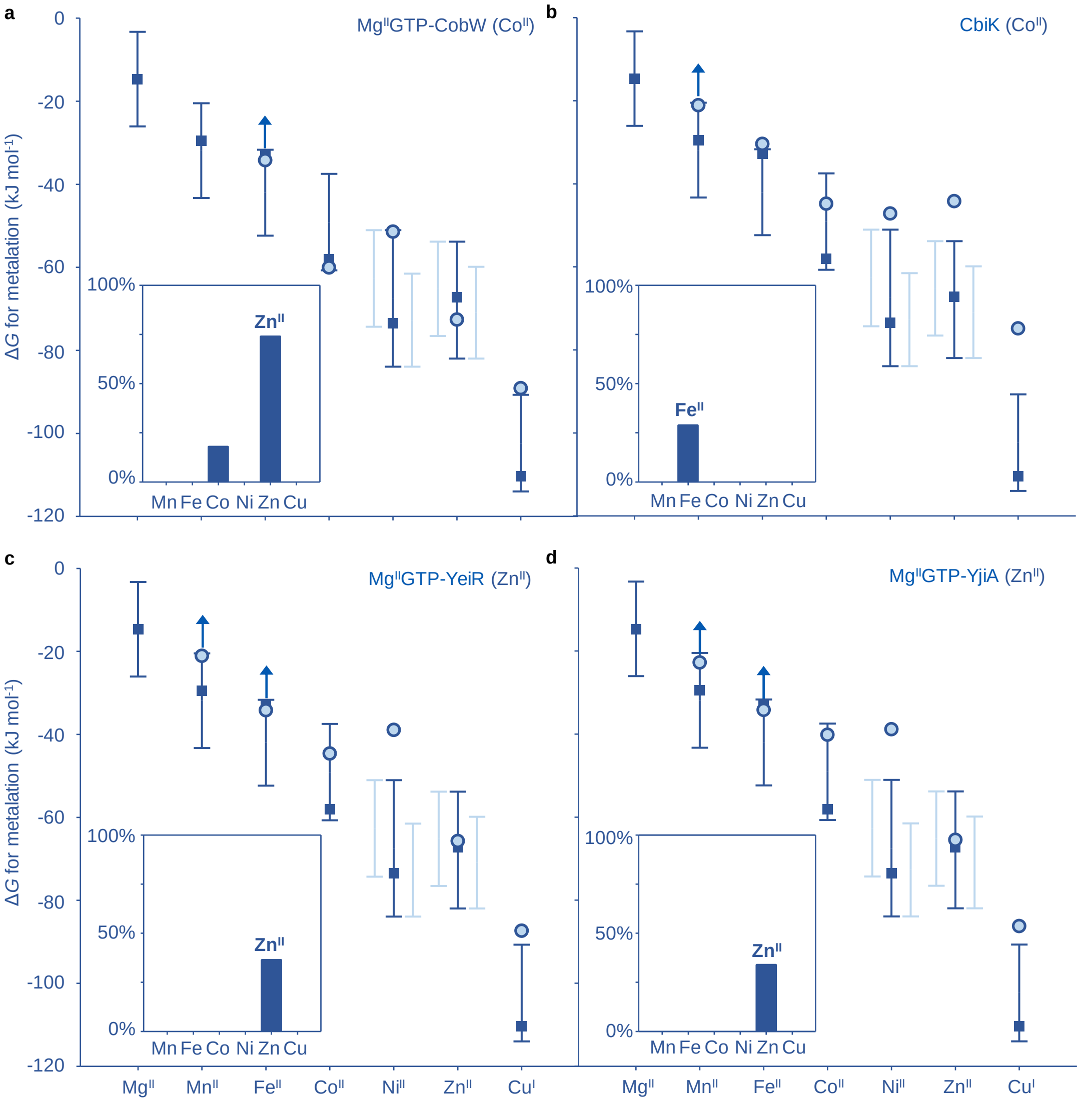


**Supplementary Fig. 20. Predicted mis-metalation of non-native proteins expressed in *E. coli*.** Free energies for metal complex formation of four exemplar proteins compared to metal availabilities in LB-grown *E. coli* as shown for MncA in Figure 4[8,14]. **a** CobW cobalt metallochaperone from *Rhodobacter* bound to $Mg^{II}$GTP, **b** CbiK cobalt chelatase from *Salmonella*, **c** YeiR putative zinc chaperone in *E. coli* and *Salmonella* in $Mg^{II}$GTP bound form[19], **d** YjiA second proposed zinc chaperone in *E. coli* and *Salmonella* in $Mg^{II}$GTP-bound state. Supplementary Data 4 was used to calculate (inset histograms) predominant mis-metalation of heterologously expressed proteins CobW and CbiK but correct metalation of *Salmonella* proteins closely related to an *E. coli* homologue. CobW is predicted to be 74% mismetalated with $Zn^{II}$ consistent with previous observations[14] (**a**), CbiK 29% predominantly mismetalated with $Fe^{II}$ using reported $K_d$ values (not $K_m$ for $Co^{II}$)[8,15] (**b**), YeiR 34% predominantly metalated with $Zn^{II}$ (**c**) and YjiA, 36% predominantly metalated with $Zn^{II}$ (**d**). Limiting affinities (indicated by small arrows), defined when the protein in question does not compete with the chosen dye in a competition assay, were excluded from the calculations. Three proteins $Mn^{II}$-MncA (Fig. 4c), $Co^{II}$-CobW and $Co^{II}$-CbiK from other organisms with no close homologues in *E. coli*, are predicted to be mis-metalated (with $Fe^{II}$, $Zn^{II}$ and $Fe^{II}$ respectively) when expressed in *E. coli*, illustrating opportunity in engineering biology to enhance yields by matching metalloenzyme preferences to metal availabilities. In all panels bars are sensor ranges, 1% to 99% MncA binding preferences (pale blue circles) and exchangeable intracellular metal availabilities (dark blue squares). Source data are provided as a Source Data file.

**Supplementary Note 1.** Derivation of calculations to formulate metal buffers with defined availabilities of two competing metals including in Supplementary Data 1.

# 1 Calculations and derivations used in constructing NTA-buffered solutions of competing metal ions.

## 1.1 Dissociation constants of NTA for divalent metal ions at pH 7.5

The affinities of nitrilotriacetate (NTA) at pH 7.5 for the divalent metals used in this study were obtained by using the absolute formation constants provided by Xiao and Wedd[7] and adjusting them to account for protonation of NTA at pH 7.5.

The Schwarzenbach α-coefficient ($\alpha$) is used to adjust the absolute formation constant of a metal ion interacting with NTA to pH 7.5.

$$\alpha = \left(1 + \beta_{H,1}[\mathrm{H}^+] + \beta_{H,2}[\mathrm{H}^+]^2 + \beta_{H,3}[\mathrm{H}^+]^3\right)^{-1}$$

Where

$$\beta_{H,1} = \frac{[\mathrm{HNTA}^{2-}]}{[\mathrm{NTA}^{3-}][\mathrm{H}^+]} = 5.37 \times 10^9$$

$$\beta_{H,2} = \frac{[\mathrm{H_2NTA}^{-}]}{[\mathrm{NTA}^{3-}][\mathrm{H}^+]^2} = 1.62 \times 10^{12}$$

$$\beta_{H,3} = \frac{[\mathrm{H_3NTA}]}{[\mathrm{NTA}^{3-}][\mathrm{H}^+]^3} = 1.26 \times 10^{14}$$

At pH 7.5, $\alpha$ = 5.85 x $10^{-3}$ for NTA.

The thermodynamic (absolute) dissociation constant for metal ion, M, from NTA is defined as:

$$K_d^M = \frac{[\mathrm{M}][\mathrm{NTA}]}{[\mathrm{M} \bullet \mathrm{NTA}]}$$

The pH-adjusted dissociation constants of NTA for divalent metal ions can then be calculated as:

$$K_d^{M\prime} = \alpha^{-1} K_d^M$$

Those values are tabulated at pH 7.5 in Supplementary Data 1.

## 1.2 Formulating NTA-buffered folding solutions of two competing metal ions

To formulate solutions containing two metal ions, M1 and M2, buffered by NTA, the starting point is to define the desired free (uncomplexed) concentration of each metal ion ([M1] and [M2]) and the total concentration of NTA ($[\mathrm{NTA}]_{tot}$) present in the folding solution.

With the above experimenter-defined parameters, it is possible to calculate the resulting concentrations of complexed metal ions ([M1•NTA] and [M2•NTA]) as follows.

First, the ratio of the concentrations of the NTA-complexes may be calculated from existing information.

$$\frac{[\mathrm{M1} \bullet \mathrm{NTA}]}{[\mathrm{M2} \bullet \mathrm{NTA}]} = \frac{[\mathrm{M1}]K_d^{M2'}}{[\mathrm{M2}]K_d^{M1'}}$$

The ratio of M2-bound NTA to free NTA concentration may also be readily determined.

$$\frac{[\mathrm{M2} \bullet \mathrm{NTA}]}{[\mathrm{NTA}]} = \frac{[\mathrm{M2}]}{K_d^{M2'}}$$

To obtain the concentration of M2•NTA,

$$[\mathrm{M2} \bullet \mathrm{NTA}] = [\mathrm{NTA}]_{\mathrm{tot}} \frac{\left({[\mathrm{M2} \bullet \mathrm{NTA}]}/{[\mathrm{NTA}]}\right)}{1 + \left({[\mathrm{M2} \bullet \mathrm{NTA}]}/{[\mathrm{NTA}]}\right)\left(1 + {[\mathrm{M1} \bullet \mathrm{NTA}]}/{[\mathrm{M2} \bullet \mathrm{NTA}]}\right)}$$

Where

$$[\mathrm{NTA}]_{\mathrm{tot}} = [\mathrm{NTA}] + [\mathrm{M1} \bullet \mathrm{NTA}] + [\mathrm{M2} \bullet \mathrm{NTA}]$$

Which allows the concentration of M1•NTA to be determined using the ratio of the complex concentrations determined above:

$$[\mathrm{M1} \bullet \mathrm{NTA}] = [\mathrm{M2} \bullet \mathrm{NTA}]\left(\frac{[\mathrm{M1} \bullet \mathrm{NTA}]}{[\mathrm{M2} \bullet \mathrm{NTA}]}\right)$$

Having already defined the desired free concentrations of M1 and M2, the total concentrations needed of each metal are calculated as:

$$[\mathrm{M1}]_{\mathrm{tot}} = [\mathrm{M1}] + [\mathrm{M1} \bullet \mathrm{NTA}]$$

and

$$[\mathrm{M2}]_{\mathrm{tot}} = [\mathrm{M2}] + [\mathrm{M2} \bullet \mathrm{NTA}]$$

These concentrations define the total amount of each metal ion stock added to the folding solution.

In formulating folding solutions, it is important to retain a significant concentration of free NTA ([NTA]) to avoid saturation. Practically, occupancy should be maintained less than 90%, which may be calculated as follows.

$$\text{Buffer occupancy} = {([\mathrm{M1} \bullet \mathrm{NTA}] + [\mathrm{M2} \bullet \mathrm{NTA}])}/{[\mathrm{NTA}]_{\mathrm{tot}}} < 90\%$$

## 2 Formulating a BCA-buffered folding solutions of $Mn^{II}$ vs. $Cu^{I}$

An experiment was performed to determine if BCA-buffered $Cu^{I}$ could compete with $Mn^{II}$ ions, which are assumed not to interact appreciably with BCA.

BCA is unprotonated at pH 7.5 [7], so the absolute formation constant for BCA with $Cu^{I}$ may be used. Note that no value for $\beta_1$ is available, indicating that the 2:1 complex of BCA with $Cu^{I}$ forms exclusively.

$$\beta_2 = \frac{[\mathrm{Cu^IBCA_2}]}{[\mathrm{Cu^I}][\mathrm{BCA}]^2} = 1.58 \times 10^{17}$$

Given the large formation constant of BCA with $Cu^I$, if the concentrations of each are in the micromolar to millimolar range, with the total concentration of BCA in excess of two-fold the total concentration of $Cu^I$ it can be assumed that

$$[\mathrm{Cu^IBCA_2}] = [\mathrm{Cu^I}]_{\mathrm{tot}}$$

Thus

$$[\mathrm{BCA}] = [\mathrm{BCA}]_{\mathrm{tot}} - 2[\mathrm{Cu^I}]_{\mathrm{tot}}$$

To determine the free, buffered concentration of $Cu^I$ ($[Cu^I]$) in such a solution, where total concentrations of $Cu^I$ and BCA are defined,

$$[\mathrm{Cu^I}] = \frac{[\mathrm{Cu^IBCA_2}]}{\beta_2[\mathrm{BCA}]^2} = \frac{[\mathrm{Cu^I}]_{\mathrm{tot}}}{\beta_2([\mathrm{BCA}]_{\mathrm{tot}} - 2[\mathrm{Cu^I}]_{\mathrm{tot}})^2}$$

**Supplementary Note 2.** Derivation of calculations to refine intracellular metal availabilities using MncA as a probe (Supplementary Data 5).

**Refining metal ion availabilities from occupancies of soluble MncA isolated from *E. coli* grown here in metal supplemented media.**

Refinement of metal ion availabilities, based on occupancies obtained for MncA purified following *in vivo* expression under supplemented growth conditions, was performed in pairwise comparisons. The metal ion whose availability is to be refined, M2, is compared to a metal ion, M1, whose availability is assumed to be at its availability in cells grown aerobically in LB.

Here, $K_{d,1}$ and $K_{d,2}$ are the pseudo-dissociation constants of MncA for M1 and M2, obtained from the preferences of MncA for M1 and M2 as described in the text.

The relative preference of MncA for M2 vs. M1 ($\mathrm{Pref}_{\mathrm{M2}}$ and $\mathrm{Pref}_{\mathrm{M1}}$, respectively) may be expressed as

$$\frac{\mathrm{Pref}_{\mathrm{M2}}}{\mathrm{Pref}_{\mathrm{M1}}} = \frac{K_{d,1}}{K_{d,2}}$$

In Table 1, the preference that MncA exhibits in trapping a metal is calculated from measured occupancies of M1 and M2 ($\mathrm{Occ}_{\mathrm{M1}}$ and $\mathrm{Occ}_{\mathrm{M2}}$) as:

$$\frac{\mathrm{Pref}_{\mathrm{M2}}}{\mathrm{Pref}_{\mathrm{M1}}} = \frac{\mathrm{Occ}_{\mathrm{M2}}}{\mathrm{Occ}_{\mathrm{M1}}} \Big/ \frac{[\mathrm{M2}]}{[\mathrm{M1}]}$$

Where [M1] and [M2] are the buffered availabilities of M1 and M2.

Thus, the availability of M2, as a buffered concentration, can be calculated as

$$[\mathrm{M2}] = [\mathrm{M1}] \frac{K_{d,2}}{K_{d,1}} \frac{\mathrm{Occ}_{\mathrm{M2}}}{\mathrm{Occ}_{\mathrm{M1}}}$$

## Supplementary References


1 Alderighi, L. *et al.* Hyperquad simulation and speciation (HySS): a utility program for the investigation of equilibria involving soluble and partially soluble species. *Coord. Chem. Rev.* **184**, 311-318 (1999).

2 Tottey, S. *et al.* Protein-folding location can regulate manganese-binding versus copper- or zinc-binding. *Nature* **455**, 1138-1142 (2008).

3 Shi, H. *et al.* Structural basis for transcription activation through cooperative recruitment of MntR. *Research Square*, rs-3 (2024).

4 Abramson, J. *et al.* Accurate structure prediction of biomolecular interactions with AlphaFold 3. *Nature* **630**, 493-500 (2024).

5 Foster, A. W., Young, T. R., Chivers, P. T. & Robinson, N. J. Protein metalation in biology. *Curr. Opin. Chem. Biol.* **66**, 102095 (2022).

6 Osman, D. & Robinson, N. J. Protein metalation in a nutshell. *FEBS Lett.* **597**, 141-150 (2023).

7 Xiao, Z. & Wedd, A. G. The challenges of determining metal–protein affinities. *Nat. Prod. Rep.* **27**, 768-789 (2010).

8 Osman, D. *et al.* Bacterial sensors define intracellular free energies for correct enzyme metalation. *Nat. Chem. Biol.* **15**, 241-249 (2019).

9 Grossoehme, N. E. & Giedroc, D. P. Energetics of allosteric negative coupling in the zinc sensor *S. aureus* CzrA. *J. Am. Chem. Soc.* **131**, 17860-17870 (2009).

10 Pennella, M. A., Arunkumar, A. I. & Giedroc, D. P. Individual metal ligands play distinct functional roles in the zinc sensor *Staphylococcus aureus* CzrA. *J. Mol. Biol.* **356**, 1124-1136 (2006).

11 Giedroc, D. P. & Arunkumar, A. I. Metal sensor proteins: nature's metalloregulated allosteric switches. *Dalton Trans.*, 3107-3120 (2007).

12 Foster, A. W. *et al.* Metalation calculators for *E. coli* strain JM109 (DE3): aerobic, anaerobic, and hydrogen peroxide exposed cells cultured in LB media. *Metallomics* **14**, mfac058 (2022).

13 Kaluarachchi, H. *et al.* Metal selectivity of the *Escherichia coli* nickel metallochaperone, SlyD. *Biochemistry* **50**, 10666-10677 (2011).

14 Young, T. R. *et al.* Calculating metalation in cells reveals CobW acquires CoII for vitamin B12 biosynthesis while related proteins prefer ZnII. *Nat. Commun.* **12**, 1195 (2021).

15 Young, T. R. *et al.* Two distinct thermodynamic gradients for cellular metalation of vitamin B12. *JACS Au* **3**, 1472-1483 (2023).

16 Blaby-Haas, C. E., Flood, J. A., de Crécy-Lagard, V. & Zamble, D. B. YeiR: a metal-binding GTPase from *Escherichia coli* involved in metal homeostasis. *Metallomics* **4**, 488-497 (2012).

17 Schneider, C. A., Rasband, W. S. & Eliceiri, K. W. NIH Image to ImageJ: 25 years of image analysis. *Nat. Methods* **9**, 671-675 (2012).

18 Tarrant, E. *et al.* Copper stress in *Staphylococcus aureus* leads to adaptive changes in central carbon metabolism. *Metallomics* **11**, 183-200 (2019).

19 Sydor, A. M. *et al.* Metal binding properties of *Escherichia coli* YjiA, a member of the metal homeostasis-associated COG0523 family of GTPases. *Biochemistry* **52**, 1788-1801 (2013).
