# Supplementary material for: A metal-trap tests and refines blueprints to engineer cellular protein metalation with different elements
Source: Nat Commun. 2025 Jan 18;16:810. doi: 10.1038/s41467-025-56199-w (PMC11742986; doi:10.1038/s41467-025-56199-w)
Supplement: Supplementary file 2 — Description of Additional Supplementary Files [file 41467_2025_56199_MOESM2_ESM.pdf]

Description of Additional Supplementary Files

Supplementary Data 1:

Calculator to formulate metal buffers with defined availabilities of two competing metals.

Supplementary Data 2:

Calculator of DNA occupancy as a function of intracellular metal availability for de-repressors.

Supplementary Data 3:

Ni^{II}RcnR refined calculator for decoding protein metal occupancies in an idealised cell.

Supplementary Data 4:

Calculator for predicting metal occupancies of proteins in aerobic *E. coli* grown in LB media.

Supplementary Data 5:

Calculator to refine intracellular metal availabilities using MncA as a probe.

Supplementary Data 6:

High Mn^{II} calculator for predicting metal occupancies of proteins in *E. coli* in LB plus Mn^{II}.

Supplementary Data 7:

High Ni^{II} calculator for predicting metal occupancies of proteins in *E. coli* in LB plus Ni^{II}.

Supplementary Data 8:

High Co^{II} calculator for predicting metal occupancies of proteins in *E. coli* in LB plus Co^{II}.

Supplementary Data 9:

Oligonucleotide sequences.

Supplementary Software (zip file):

Dynafit scripts to describe competition between EGTA and RcnR for NiII and NiII - bound holo-RcnR tetramers binding to rcnRA.
