# Supplementary material for: A metal-trap tests and refines blueprints to engineer cellular protein metalation with different elements
Source: Nat Commun. 2025 Jan 18;16:810. doi: 10.1038/s41467-025-56199-w (PMC11742986; doi:10.1038/s41467-025-56199-w)
Supplement: Supplementary file 12 — Supplementary Software [file 41467_2025_56199_MOESM12_ESM.zip › Supplemental Software - Copy.docx]

**Supplementary Software**

**Dynafit Scripts**

1. **Dynafit script to describe competition between EGTA and RcnR for Ni^II^**

Used to generate Figure 2b and Supplementary Figure 3b. An average dissociation constant was calculated for the allosterically induced RcnR tetramer with two bound metal ions (K_1_). Two Ni^II^ additions exceeded the saturation of EGTA and are excluded from the fit.

[model]

RcnR binds one Ni per subunit, EGTA binds one Ni. Binding to RcnR tetramer is stepwise.

[components]

; all concentrations in micromolar

; P = RcnR tetramer

; N = Ni

; E = EGTA

[task]

task = fit

data = equilibria

[mechanism]

P + N <==> PN : K1a dissociation

PN + N <==> PN2 : K1b dissociation

PN2 + N <==> PN3 : K1c dissociation

PN3 + N <==> PN4 : K1d dissociation

E + N <==> EN : Keq2 dissociation

[constants]

K1a = 0.000007 ?

K1b = 2.67 * K1a

K1c = 6 * K1a

K1d = 16 * K1a

Keq2 = 5.15e-5

[responses]

PN = 0.25 * PN4

PN2 = 0.5 * PN4

PN3 = 0.75 * PN4

PN4 = .0022 ?

[data]

variable N

set rcnr1 | concentration P = 3.825, E = 243

set rcnr2 | concentration P = 6.325, E = 479

set rcnr3 | concentration P = 7.875, E = 471

set rcnr4 | concentration P = 10.1, E = 464

[output]

directory B:\Figures

[set:rcnr1]

0 0

6.674872993 0.000743

11.12478832 0.001402

18.54131387 0.00237

30.90218978 0.003515

51.50364964 0.004562

85.83941606 0.005914

143.0656934 0.006585

;238.4428224 0.008843

193.7347932 0.006313

;272.9407485 0.008178

[set:rcnr2]

0 0

7.885143377 0.001333

13.14190563 0.001883

21.90317605 0.003748

36.50529341 0.004695

60.84215569 0.007059

101.4035928 0.009279

169.005988 0.010206

281.6766467 0.010688

;469.4610778 0.014264

375.5688623 0.01054

;519.884522 0.013908

[set:rcnr3]

0 0

7.913575385 0.001071

13.18929231 0.002668

21.98215385 0.003842

36.63692308 0.005393

61.06153846 0.008242

101.7692308 0.011258

169.6153846 0.013674

282.6923077 0.013619

;471.1538462 0.019525

376.9230769 0.01514

;547.0583783 0.020859

[set:rcnr4]

0 0

7.801060095 0.001112

13.00176682 0.001991

21.66961137 0.003684

36.11601896 0.006533

60.19336493 0.009373

100.3222749 0.013998

167.2037915 0.017199

278.6729858 0.017614

;464.4549763 0.025375

371.563981 0.020015

;541.6992824 0.027549

[end]

**2. Dynafit script to describe Ni^II^-bound holo-RcnR tetramers binding to *rcnRA***

Used to generate Figure 2c and to estimate K_4_.

[model]

Two RcnR tetramers bind independently to two sites on *rcnRA* promoter in a stepwise fashion. Assay performed at pH 7.5.

[components] ;concentrations in nanomolar

; P = RcnRtetramer

; D = DNA containing rcnRA sequence

[task]

task = fit

data = equilibria

[mechanism]

P + D <==> P.D : K1 dissoc

P + P.D <==> P2.D : K2 dissoc

[constants] ;nanomolar

K1 = 10 ?

K2 = 4 * K1

[responses]

P.D = 0.5 * P2.D

P2.D = 0.01115 ; fixed response Osman et al. (2016)

[data]

variable P

plot logarithmic

set DNA1 | concentration D = 10

set DNA2 | concentration D = 10

set DNA3 | concentration D = 10

[output]

directory B:\Figures

[set:DNA1]

0 0

5 -0.000866667

10 -0.001016667

20 -0.000216667

30 0.000933333

40 -0.001066667

50 0.000683333

75 -0.000666667

100 0.000833333

150 0.004233333

200 0.004283333

250 0.005233333

375 0.007033333

500 0.006683333

750 0.012533333

1000 0.015133333

1250 0.016733333

1750 0.023633333

2500 0.031633333

3750 0.039533333

5000 0.052733333

7500 0.059883333

10000 0.072683333

[set:DNA2]

0 0

5 -0.00095

10 -0.00015

20 0.0012

30 -0.0016

40 -0.00125

50 -0.0013

75 0.00205

100 0.0025

150 0.0055

200 0.0035

250 0.0033

375 0.00505

500 0.00775

750 0.00915

1000 0.01415

1250 0.01835

1750 0.02285

2500 0.03125

3750 0.0419

5000 0.0482

7500 0.0616

10000 0.07355

[set:DNA3]

0 0

5 0.001233333

10 -0.001916667

20 0.000983333

30 -0.000266667

40 0.000283333

50 0.000783333

75 -0.001366667

100 0.001583333

150 0.003633333

200 0.003833333

250 0.005233333

375 0.009433333

500 0.006983333

750 0.011783333

1000 0.012883333

1250 0.018733333

1750 0.023633333

2500 0.032033333

3750 0.042133333

5000 0.050983333

7500 0.061733333

10000 0.069133333

[end]
