# Supplementary material for: A metal-trap tests and refines blueprints to engineer cellular protein metalation with different elements
Source: Nat Commun. 2025 Jan 18;16:810. doi: 10.1038/s41467-025-56199-w (PMC11742986; doi:10.1038/s41467-025-56199-w)
Supplement: Supplementary file 13 — Reporting Summary [file 41467_2025_56199_MOESM13_ESM.pdf]

nature portfolio

Corresponding author(s): Nigel J Robinson

Last updated by author(s): Dec 18, 2024

Reporting Summary

Nature Portfolio wishes to improve the reproducibility of the work that we publish. This form provides structure for consistency and transparency in reporting. For further information on Nature Portfolio policies, see our Editorial Policies and the Editorial Policy Checklist.

Statistics

For all statistical analyses, confirm that the following items are present in the figure legend, table legend, main text, or Methods section.

n/a	Confirmed	
☐	☒	The exact sample size (*n*) for each experimental group/condition, given as a discrete number and unit of measurement
☐	☒	A statement on whether measurements were taken from distinct samples or whether the same sample was measured repeatedly
☒	☐	The statistical test(s) used AND whether they are one- or two-sided<br>*Only common tests should be described solely by name; describe more complex techniques in the Methods section.*
☒	☐	A description of all covariates tested
☒	☐	A description of any assumptions or corrections, such as tests of normality and adjustment for multiple comparisons
☐	☒	A full description of the statistical parameters including central tendency (e.g. means) or other basic estimates (e.g. regression coefficient) AND variation (e.g. standard deviation) or associated estimates of uncertainty (e.g. confidence intervals)
☒	☐	For null hypothesis testing, the test statistic (e.g. *F*, *t*, *r*) with confidence intervals, effect sizes, degrees of freedom and *P* value noted<br>*Give P values as exact values whenever suitable.*
☒	☐	For Bayesian analysis, information on the choice of priors and Markov chain Monte Carlo settings
☒	☐	For hierarchical and complex designs, identification of the appropriate level for tests and full reporting of outcomes
☒	☐	Estimates of effect sizes (e.g. Cohen's *d*, Pearson's *r*), indicating how they were calculated

Our web collection on statistics for biologists contains articles on many of the points above.

Software and code

Policy information about availability of computer code

Data collection	Crystallographic data were collected and analysed at beamline I04, Diamond Light Source; Agilent Cary 3500 and Perkin-Elmer lambda 35 were used for UV-vis spectroscopy; Cary Eclipse Version 1.1 Scan Application was used for fluorescence spectroscopy; BioRad ChemiDoc MP was used to collect images of SDS-PAGE gels; cell volumes were determined using the Casy Cell counter (TT, Innovatis); Rotor-Gene Q-Pure Detection Version 2.3.4 (Qiagen) was used to collect qPCR data; Plasma Lab Version 2.6.2.337 (ThermoFisher) and iCAP RQ (ThermoFisher) Qtegra version 2.14.5122.306 was used to collect ICP-MS data.
Data analysis	ChimeraX 1.7, Phenix 1.20, PyMOL 2.3.5, Coot 0.9.8.93 and Mole 2.5 were used to produce and analyse the MncA-Ni structure model; Sigma Plot Version 14.0 was used to produce graphs; Dynafit Version 4 (BioKin Ltd) was used to fit biochemical data (example scripts provided in Supplementary Software); ImageJ 1.53 was used to measure band density in gels and R software 4.4.1 was used in principal component analysis; Lin Reg PCR Version 2021.1 was used to analyze qPCR data; MATLAB R2020b Version 9.9.0.1538559 was used to determine buffered metal concentration from DNA occupancy of sensors (using code from Main Text ref. 23); Microsoft Excel Version 2402 was used to produce the metal buffer and metalation calculator spreadsheets and report the oligonucleotide sequences (Supplementary Data 1-9).

For manuscripts utilizing custom algorithms or software that are central to the research but not yet described in published literature, software must be made available to editors and reviewers. We strongly encourage code deposition in a community repository (e.g. GitHub). See the Nature Portfolio guidelines for submitting code & software for further information.

Data

Policy information about availability of data

All manuscripts must include a data availability statement. This statement should provide the following information, where applicable:

- Accession codes, unique identifiers, or web links for publicly available datasets
- A description of any restrictions on data availability
- For clinical datasets or third party data, please ensure that the statement adheres to our policy

A data availability statement is included: "All data are available within the article, its Supplementary Information files, plus PDB entry 9GOF (https://doi.org/10.2210/pdb9GOF/pdb) and from corresponding authors on request. Source data are provided with this paper as Source Data files. Excel spreadsheets (with instructions) providing a calculator to formulate competing metal buffers, to calculate DNA occupancy as a function of NiII availability for metal-dependent de-repressor RcnR and providing a calculator to use in vivo recovered metal occupancies of MncA as a probe to refine estimates of intracellular metal availabilities, are provided as Supplementary Data 1, 2 and 5 respectively. Excel spreadsheets constituting calculators of metalation in NiII-RcnR-refined idealised cells, E. coli grown aerobically in LB, E. coli grown aerobically in LB supplemented with manganese, nickel, and cobalt, are provide as Supplementary Data 3, 4, 6-8 respectively. Supplementary Data 9 contains oligonucleotide sequences. Published structures used here for MncA and MntR are PDB entries 2VQA (https://doi.org/10.2210/pdb2VQA/pdb) and 9C4D (https://doi.org/10.2210/pdb9C4D/pdb) respectively."

Research involving human participants, their data, or biological material

Policy information about studies with human participants or human data. See also policy information about sex, gender (identity/presentation), and sexual orientation and race, ethnicity and racism.

Reporting on sex and gender	*Use the terms sex (biological attribute) and gender (shaped by social and cultural circumstances) carefully in order to avoid confusing both terms. Indicate if findings apply to only one sex or gender; describe whether sex and gender were considered in study design; whether sex and/or gender was determined based on self-reporting or assigned and methods used. Provide in the source data disaggregated sex and gender data, where this information has been collected, and if consent has been obtained for sharing of individual-level data; provide overall numbers in this Reporting Summary. Please state if this information has not been collected. Report sex- and gender-based analyses where performed, justify reasons for lack of sex- and gender-based analysis.*
Reporting on race, ethnicity, or other socially relevant groupings	*Please specify the socially constructed or socially relevant categorization variable(s) used in your manuscript and explain why they were used. Please note that such variables should not be used as proxies for other socially constructed/relevant variables (for example, race or ethnicity should not be used as a proxy for socioeconomic status). Provide clear definitions of the relevant terms used, how they were provided (by the participants/respondents, the researchers, or third parties), and the method(s) used to classify people into the different categories (e.g. self-report, census or administrative data, social media data, etc.) Please provide details about how you controlled for confounding variables in your analyses.*
Population characteristics	*Describe the covariate-relevant population characteristics of the human research participants (e.g. age, genotypic information, past and current diagnosis and treatment categories). If you filled out the behavioural & social sciences study design questions and have nothing to add here, write "See above."*
Recruitment	*Describe how participants were recruited. Outline any potential self-selection bias or other biases that may be present and how these are likely to impact results.*
Ethics oversight	*Identify the organization(s) that approved the study protocol.*

Note that full information on the approval of the study protocol must also be provided in the manuscript.

Field-specific reporting

Please select the one below that is the best fit for your research. If you are not sure, read the appropriate sections before making your selection.

☒ Life sciences ☐ Behavioural & social sciences ☐ Ecological, evolutionary & environmental sciences

For a reference copy of the document with all sections, see nature.com/documents/nr-reporting-summary-flat.pdf

Life sciences study design

All studies must disclose on these points even when the disclosure is negative.

Sample size	Sample sizes were chosen based on prior experimental experience, and to give consistent results, following convention in the literature for equivalent analyses (eg, ref. 22 and 23 in main manuscript). Experiments designed to derive quantitative values used to model or test calculations of metalation were performed in triplicate (n=3) to enable calculation of SD (listed in Tables or shown as error bars in Figures) with an additional replicate (to n=4) performed to test an additional nickel to manganese ratio to ensure triplicated data had not fallen below reliable detection thresholds. Replication of growth in un-supplemented medium exceeded n= 3 (n=12) because this control was repeated in multiple analyses effects of different metals. Single replicates (n=1) were only used qualitatively where analyses were not used to model or test calculations of metalation. SE used was used for simultaneously fitting binding isotherms for RcnR, and n=4 independent titrations of nickel with different ratios of competing EGTA. The number of independent experiments or biologically independent samples is shown in

	Figure legends or footnotes of Tables.
Data exclusions	Competitions between nickel and manganese without histidine are shown but not propagated into binding preferences to avoid interference from any unaccounted competing molecules. Fitting of 4 independent RcnR nickel-titrations excluded 2 values in 1 titration where EGTA was saturated. qPCR replicates do not vary by more than 2 cycles except 3 values noted in the Source Data TXT file, hence excluded from subsequent analyses. Exclusion criteria were pre-established.
Replication	Replicates ('n'), and their nature (n always refers to independent experiments and/or biologically independent samples, not technical replicates) are specifically defined in the Figure legends or Table footnotes and in the Statistics and Reproducibility section of the materials and methods.
Randomization	Data collection by instruments (absorbance, fluorescence, ICP-MS, qPCR, image capture and analysis, cell-counting) was independent from the experimenter when collected therefore randomisation was not performed. Bacterial colonies used for protein purification, and gene expression analyses were chosen at random.
Blinding	No blinding was used in this study as the types of measurements made (see randomisation) were not susceptible to subjective bias.

Reporting for specific materials, systems and methods

We require information from authors about some types of materials, experimental systems and methods used in many studies. Here, indicate whether each material, system or method listed is relevant to your study. If you are not sure if a list item applies to your research, read the appropriate section before selecting a response.

Materials & experimental systems

n/a	Involved in the study
☒	☐ Antibodies
☒	☐ Eukaryotic cell lines
☒	☐ Palaeontology and archaeology
☒	☐ Animals and other organisms
☒	☐ Clinical data
☒	☐ Dual use research of concern
☒	☐ Plants

Methods

n/a	Involved in the study
☒	☐ ChIP-seq
☒	☐ Flow cytometry
☒	☐ MRI-based neuroimaging

Plants

Seed stocks	*Report on the source of all seed stocks or other plant material used. If applicable, state the seed stock centre and catalogue number. If plant specimens were collected from the field, describe the collection location, date and sampling procedures.*
Novel plant genotypes	*Describe the methods by which all novel plant genotypes were produced. This includes those generated by transgenic approaches, gene editing, chemical/radiation-based mutagenesis and hybridization. For transgenic lines, describe the transformation method, the number of independent lines analyzed and the generation upon which experiments were performed. For gene-edited lines, describe the editor used, the endogenous sequence targeted for editing, the targeting guide RNA sequence (if applicable) and how the editor was applied.*
Authentication	*Describe any authentication procedures for each seed stock used or novel genotype generated. Describe any experiments used to assess the effect of a mutation and, where applicable, how potential secondary effects (e.g. second site T-DNA insertions, mosiacism, off-target gene editing) were examined.*
